# Spatiotemporal processing of neural cell adhesion molecules 1 and 2 by BACE1 *in vivo*

**DOI:** 10.1016/j.jbc.2021.100372

**Published:** 2021-02-03

**Authors:** WonHee Kim, Hiroto Watanabe, Selene Lomoio, Giuseppina Tesco

**Affiliations:** Alzheimer's Disease Research Laboratory, Department of Neuroscience, Tufts University School of Medicine, Boston, Massachusetts, USA

**Keywords:** BACE1, β-secretase, neural cell adhesion molecule 1 (NCAM1), neural cell adhesion molecule 2 (NCAM2), polysialic acid (PSA), synaptosome, substrate, Aβ, amyloid β peptides, AD, Alzheimer's disease, ADAM, a disintegrin and metalloprotease, ANOVA, analysis of variance, APLP1, amyloid precursor protein-like protein 1, APLP2, amyloid precursor protein-like protein 2, APP, amyloid precursor protein, Asn, Asparagine, Asp, aspartic acid, BACE1, β-site amyloid precursor protein cleaving enzyme 1, CACHD1, VWFA and cache domain-containing protein 1, CHL1, cell adhesion molecule L1 like, cKO, conditional knockout, CM, conditioned media, CTF, C-terminal fragment, DMEM, Dulbecco's modified Eagles medium, ECMs, extracellular matrix molecules, EV, empty vector, FBS, fetal bovine serum, FN, fibronectin type III, GI, GI254023X, Glu, glutamic acid, GM, GM6001, GPI, glycosylphosphatidylinositol, h, human, HC, hippocampus, HRP, horseradish peroxidase, Ig, immunoglobulin-like domains, IgSF-CAMs, cell adhesion molecules of the immunoglobulin superfamily, IPB, infra pyramidal bundles, Jag1, Jagged 1, LC/MS/MS, microcapillary tandem liquid chromatography tandem mass spectrometry, LPT, long-term potentiation, mAb, monoclonal antibody, MDGA1, MAM domain-containing glycosylphosphatidylinositol anchor protein 1, MMP, matrix metalloproteinase, NCAM1, neural cell adhesion molecule 1, NCAM2, neural cell adhesion molecule 2, non-PSA-NCAM1, nonpolysialylated NCAM1, NTF, N-terminal fragment, OB, olfactory bulb, OSNs, olfactory sensory neurons, P10, postnatal day 10, pAb, polyclonal antibody, PSA-NCAM1, polysialylated NCAM1, PSA, polysialic acid, PSD95, postsynaptic protein marker, PVP-40, Polyvinyl-pyrrolidone, RIPA, radioimmunoprecipitation assay, SEZ6, Seizure protein 6, SEZ6L, Seizure 6-like protein, sNCAM1, soluble NCAM1 fragments, sNCAM2, soluble NCAM2 fragments, SPB, suprapyramidal bundles, ST3Gal, beta-galactoside alpha-2,3-sialyltransferase, ST6Gal, beta-galactoside alpha-2,6-sialyltransferase, TM, transmembrane, VGSCβ, β-subunits of voltage-gated sodium channel, WT, wild type

## Abstract

Neural cell adhesion molecules 1 (NCAM1) and 2 (NCAM2) belong to the cell adhesion molecules of the immunoglobulin superfamily and have been shown to regulate formation, maturation, and maintenance of synapses. NCAM1 and NCAM2 undergo proteolysis, but the identity of all the proteases involved and how proteolysis is used to regulate their functions are not known. We report here that NCAM1 and NCAM2 are BACE1 substrates *in vivo*. NCAM1 and NCAM2 overexpressed in HEK cells were both cleaved by metalloproteinases or BACE1, and NCAM2 was also processed by γ-secretase. We identified the BACE1 cleavage site of NCAM1 (at Glu 671) and NCAM2 (at Glu 663) using mass spectrometry and site-directed mutagenesis. Next, we assessed BACE1-mediated processing of NCAM1 and NCAM2 in the mouse brain during aging. NCAM1 and NCAM2 were cleaved in the olfactory bulb of BACE1+/+ but not BACE1−/− mice at postnatal day 10 (P10), 4 and 12 months of age. In the hippocampus, a BACE1-specific soluble fragment of NCAM1 (sNCAM1β) was only detected at P10. However, we observed an accumulation of full-length NCAM1 in hippocampal synaptosomes in 4-month-old BACE1−/− mice. We also found that polysialylated NCAM1 (PSA-NCAM1) levels were increased in BACE1−/− mice at P10 and demonstrated that BACE1 cleaves both NCAM1 and PSA-NCAM1 *in vitro*. In contrast, we did not find evidence for BACE1-dependent NCAM2 processing in the hippocampus at any age analyzed. In summary, our data demonstrate that BACE1 differentially processes NCAM1 and NCAM2 depending on the region of brain, subcellular localization, and age *in vivo*.

Alzheimer's disease (AD) is pathologically defined by the presence of extracellular amyloid plaques, mainly constituted by amyloid β peptides (Aβ), along with intracellular neurofibrillary tangles composed of hyperphosphorylated tau (1, 2). A large number of studies suggests that excessive cerebral accumulation of Aβ plays a central role in the development of AD pathology. The amyloid precursor protein (APP) can be cleaved by BACE1 at Asp1 (β-site; the first N-terminal residue of Aβ in APP) or at Glu11 (β′-site), generating soluble N-terminal APP fragments (sAPPβ or sAPPβ′, respectively) and membrane-bound C-terminal APP fragments (C99 or C89, respectively) (3, 4, 5, 6). Subsequent cleavage of C99 by γ-secretase generates neurotoxic Aβ (7, 8). Therefore, BACE1 has been considered a prime therapeutic drug target for lowering Aβ levels in the AD brain. However, clinical trials based on BACE inhibitors verubecestat and atabecestat were discontinued due to a modest worsening of cognitive function and other adverse effects including the onset of psychiatric symptoms (9, 10, 11, 12, 13). More recently, Biogen and Eisai discontinued two phase-III trials (with BACE inhibitor elenbecestat) due to an unfavorable risk–benefit ratio. Given that the different molecules produced similar adverse effects, most likely these are mechanism-based side effects owing to reduced processing of BACE1 substrates that are essential for normal neuronal function. In order to understand and possibly avoid the mechanism-based side effects caused by BACE inhibitors, it is important to identify and validate BACE1 substrates *in vivo*.

Besides APP, more than 60 putative BACE1 substrates have been reported *in vitro* using proteomic analysis (14, 15, 16, 17). Among them, only a few have been validated to date as BACE1 substrates *in vivo*, including neuregulin-1, Jagged 1 (Jag1), β-subunits of voltage-gated sodium channel (VGSCβ), amyloid precursor protein-like protein 1 and 2 (APLP1, APLP2), seizure protein 6 (SEZ6), seizure 6-like protein (SEZ6L), L1, cell adhesion molecule L1 like (CHL1), VWFA and cache domain-containing protein 1 (CACHD1), and MAM domain-containing glycosylphosphatidylinositol anchor protein 1 (MDGA1) (14, 18, 19, 20, 21, 22, 23, 24, 25, 26).

Neural cell adhesion molecule 1 (NCAM1) and neural cell adhesion molecule 2 (NCAM2) belong to the cell adhesion molecules (CAMs) of the immunoglobulin superfamily (IgSF-CAMs). The overall structure of NCAM1 and NCAM2 is similar: five immunoglobulin-like domains (Ig 1–5) and two fibronectin type III repeats (FN 1–2). NCAM2 is expressed as a transmembrane isoform with a cytosolic region (NCAM2-TM) or a glycosylphosphatidylinositol (GPI)-anchored (NCAM2-GPI) isoform (Fig. 1*A*). Similarly, three isoforms of NCAM1 are produced: two transmembrane isoforms with cytoplasmic tails of different lengths (NCAM1-180 or NCAM1-140) and a GPI-anchored isoform (NCAM1-120) (Fig. 1*B*). NCAM2-TM and NCAM1-140 amino acid sequences are ∼44% identical (27). NCAM2 contains eight putative Asn-linked glycosylation sites (N-glycosylation), while NCAM1 has six N-glycosylation sites (Fig. 1*C*, blue triangles). NCAM1 is known to be posttranslationally modified by a linear chain of alpha 2,8 linked 5-N-acetylneuraminic acid of variable length (from 8 to 400), also known as polysialic acid (PSA) (28, 29). In the fifth Ig domain (Ig5) of NCAM1, two of the three N-glycosylation sites are polysialylated (Fig. 1*C*, right panel). Sialic acid molecules are added to α-2,3 or α-2,6-linked mono-sialylated NCAM1 by two Golgi-resident polysialyltransferase ST8SIA2 (also known as STX) and ST8SIA4 (also known as PST) (29). ST8SIA2 is mainly responsible for PSA modification of NCAM1 in the developing brain, whereas ST8SIA4 is the major polysialyltransferase of the adult brain (30, 31, 32, 33).

**Figure 1:**
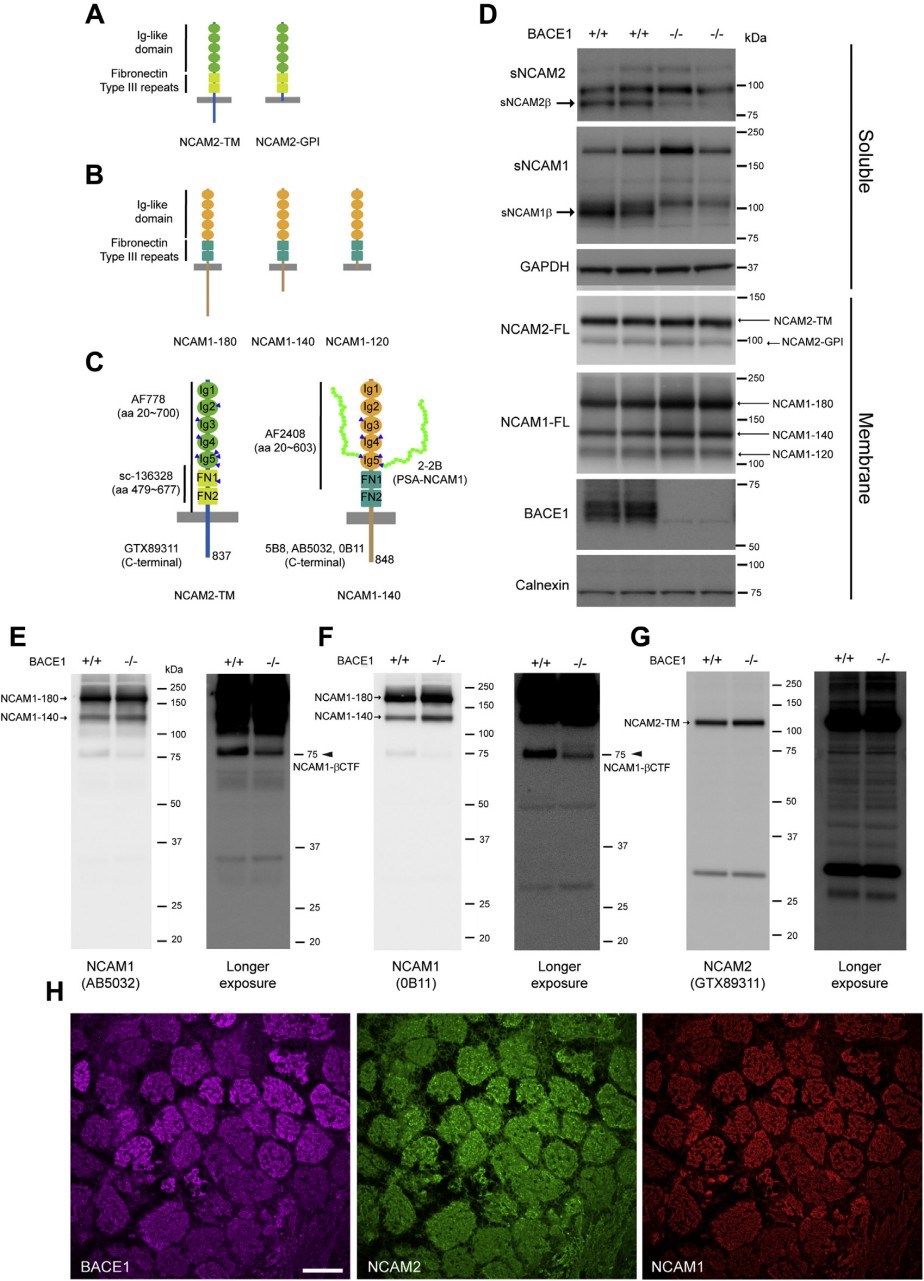
**NCAM2 and NCAM1 are BACE1 substrates in the mouse olfactory bulb.***A–B*, schematic diagram of the two mouse NCAM2 (transmembrane [TM] and GPI-anchored) isoforms (*A*) and three major mouse NCAM1 (transmembrane [NCAM1-180 and NCAM1-140] and GPI-anchored [NCAM1-120]) isoforms (*B*) resulting from alternative splicing. NCAM2 and NCAM1 have five immunoglobulin (Ig)-like domains represented by ovals and two fibronectin type III repeats (FN) represented by rectangles. *C*, schematic presentation of NCAM2-TM and polysialylated NCAM1-140 illustrating antibody-binding sites. NCAM2 contains eight putative Asn-linked glycosylation sites (N-glycosylation) indicated by blue triangles, while NCAM1 has six N-glycosylation sites. In the fifth Ig (Ig5) of NCAM1, two of three N-glycosylation sites are polysialylated, represented by a chain of green circles (sialic acid). *D*, representative immunoblot of PBS soluble fraction (Soluble) and membrane fraction (Membrane) of olfactory bulb samples from 4-month-old BACE1+/+ and BACE1−/− mice using anti-NCAM2 (sc-136328), anti-NCAM1 (AF-2408), BACE1 (D10E5), GAPDH (MAB374), and anti-calnexin (610523) antibodies. BACE1-specific soluble NCAM2 (sNCAM2β) and NCAM1 (sNCAM1β) are observed in BACE1+/+ mice, but not in BACE1−/− mice. Full-length NCAM1 (NCAM1-180 and NCAM1-140, but not NCAM1-120) levels were slightly increased in BACE1−/−, compared with BACE1+/+ mice. However, levels of full-length NCAM2 (NCAM2-TM and NCAM2-GPI) were similar in BACE1+/+ and BACE1−/− mice. *E–G*, representative immunoblot of membrane fractions of olfactory bulb samples from 4-month-old BACE1+/+ and BACE1−/− mice using two different anti-C-terminal NCAM1 (rabbit pAb; AB5032 [*E*] and mouse mAb; 0B11 [*F*]) and anti-C-terminal NCAM2 (goat pAb; GTX89311 [*G*]) antibodies. After longer exposure, a ∼75 kDa NCAM1-CTF was detected in BACE1+/+ mice, but it was greatly decreased in BACE1−/− mice, and thus termed NCAM1-βCTF (arrow in *D* and *E*). However, a BACE1-specific NCAM2-βCTF was not observed (*G*). *H*, sagittal section from 12-month-old BACE1+/+ olfactory bulb was stained with anti-BACE1 (3D5, *magenta*), anti-NCAM2 (AF778, *green*), and anti-NCAM1 (AB5032, *red*) antibodies. NCAM1 and NCAM2 immunostaining with BACE1 are significantly overlapped in glomeruli. Scale bar represents 100 μm. *D* (BACE1+/+; n = 5–6, BACE1−/−; n = 5); *E–G* (BACE1+/+; n = 3 and BACE1−/−; n = 3); *H* (BACE1+/+; n = 4, two sagittal (*H*) and two coronal [Fig. S3] sections).

Polysialylated NCAM1 (PSA-NCAM1) is predominantly found in the embryonic brain (27, 34), and its expression in the adult brain is highly restricted to the brain regions where neurogenesis is ongoing such as the hippocampus, subventricular zone, and olfactory system (34, 35, 36). Therefore, the polysialylation of NCAM1 needs to be highly regulated for normal cellular functions. In contrast, NCAM2 is not modified with PSA, suggesting that NCAM1 and NCAM2 have distinctive biological function *in vivo* (27).

NCAMs have been shown to regulate formation, maturation, and maintenance of synapses (37). Since synaptic loss is one of the earliest signs of AD (38), the relationship between NCAMs and AD has been described in a number of reviews (39, 40). Reduced synaptic levels of NCAM1 were reported in the frontal and temporal cortex of AD brains (41). Similarly, Leshchyns'ka *et al*. (42) showed that the levels of synaptic NCAM2 were significantly reduced while levels of soluble fragment of NCAM2 were increased in the hippocampus of AD brains compared with controls. These findings suggest an increased processing of NCAM2 at synapses. However, the protease responsible for NCAM2 processing has not been identified. Because BACE1 levels and activity are increased in AD brains compared with controls (43, 44, 45), we hypothesized that NCAM2 is a BACE1 substrate. In contrast, the proteolysis of NCAM1 by metalloproteinases, including a disintegrin and metalloprotease (ADAM) 10, ADAM17, matrix metalloproteinase (MMP)-2, and MMP-9, has been well characterized (46, 47, 48, 49). Moreover, proteomic studies reported that NCAM1 is a putative BACE1 substrate *in vitro* (15, 17). However, the cleavage of NCAM1 by BACE1 was not previously validated *in vivo*.

Here, we report that NCAM1 and NCAM2 are BACE1 substrates *in vivo*. We demonstrate that BACE1 differentially processes NCAM1 and NCAM2 depending on the region of brain, subcellular localization, and age *in vivo*. Our data indicate that BACE1 controls neuronal physiology by spatiotemporally regulating the proteolysis of some of its substrates.

## Results

### NCAM2 and NCAM1 are BACE1 substrates in the mouse olfactory bulb

Since NCAM2 is highly expressed in mouse olfactory epithelium and the brain (27), we first investigated the proteolysis of NCAM2 in the olfactory bulb (OB) of BACE1+/+ and BACE1−/− mice at the age of 4 months. Sequentially prepared soluble and membrane fractions were analyzed by immunoblot with mouse anti-N-terminal NCAM2 antibody (sc-136328) (Fig. 1*C*). Three soluble NCAM2 fragments (sNCAM2) were detected in the soluble fraction of BACE1+/+ OB (Fig. 1*D*). Importantly, an sNCAM2 fragment with a size of ∼88 kDa was almost absent in BACE1−/− samples (Fig. 1*D*, arrow), indicating that it is generated by BACE1 cleavage (sNCAM2β). To confirm the specificity of the BACE1-derived fragment, western blot was performed with a goat anti-N-terminal NCAM2 antibody (AF778) previously validated in NCAM2 null tissue (Fig. S1*B* and Table S1). Consistent with the data obtained with sc-136328 mouse antibody (Figs. 1*C* and S1*A*), an sNCAM2 fragment with a size of ∼88 kDa was almost absent in BACE1−/− samples (Fig. S1*B*, arrow), indicating that it is generated by BACE1 cleavage (sNCAM2β). In order to identify nonspecific bands detected by secondary antibodies, we performed western blot with HRP-conjugated secondary antibody (anti-mouse and anti-goat) without primary antibodies (Fig. S1*D*). Although nonspecific bands were detected (open arrowhead), none of these bands corresponded to the ∼88 kDa sNCAM2 fragment (Fig. S1, *B* and *D*). In the membrane fraction of BACE1+/+ and BACE1−/− OB, two bands corresponding to the two isoforms of NCAM2 (NCAM2-TM and NCAM2-GPI) were detected (Fig. 1, *A* and *D*). However, there was no change in the levels of full-length NCAM2 (NCAM2-FL) in BACE1−/− OB (Fig. 1*D*).

Next, we assessed the proteolysis of NCAM1 in soluble and membrane fractions, prepared from OB of BACE1+/+ and BACE1−/− mice. Immunoblot analysis with goat anti-N-terminal NCAM1 antibody (AF2408) (Fig. 1*C*) revealed three soluble NCAM1 fragments (sNCAM1) in the soluble fraction of BACE1+/+ OB (Fig. 1*D*). Similar to NCAM2, an sNCAM1 fragment with a size of ∼95 kDa was not detected in BACE1−/− samples (Fig. 1*D*, arrow), indicating that it is generated by BACE1 cleavage (sNCAM1β). Western blot with HRP-conjugated anti-goat secondary antibody without the primary antibody revealed nonspecific bands (Fig. S1*C*, open arrowhead). However, none of these bands corresponded to the ∼95 kDa sNCAM1 fragment. Three isoforms of full-length NCAM1 (NCAM1-120, NCAM1-140, and NCAM1-180) were detected in the membrane fraction of BACE1+/+ OB (Fig. 1*D*). A slight increase in the levels of full-length NCAM1 (NCAM1-180 and NCAM1-140, but not NCAM1-120) was observed in BACE1−/− samples compared with BACE+/+ samples (Fig. 1, *A* and *D*).

In order to detect BACE1-specific C-terminal NCAM1 and NCAM2 fragment (NCAM1-βCTF and NCAM2-βCTF, respectively) *in vivo*, we performed western blot of membrane fraction of 4-month-old BACE1+/+ and BACE1−/− OB samples using anti-C-terminal NCAM1 (rabbit pAb; AB5032 and mouse mAb; 5B8) and anti-C-terminal NCAM2 (goat pAb; GTX89311) antibodies (Fig. 1, *E*–*G*). Full-length NCAM1-180 and NCAM1-140 were detected by three different anti-C-terminal NCAM1 antibodies (AB5032, 0B11, and 5B8), while GPI-anchored NCAM1-120 was not detected owing to the absence of C-terminal cytoplasmic domain (Fig. 1, *B*, *E*, and F, and Fig S2*A*, arrow). All the anti-C-terminal NCAM1 antibodies detected a ∼75 kDa C-terminal NCAM1 fragment (NCAM1-βCTF) that was significantly decreased in BACE1−/− OB samples, indicating that it is produced by BACE1 cleavage (NCAM1-βCTF) (Fig. 1, *E* and *F*, and Fig S2*A*, arrowhead). The specificity of both AB5032 and 5B8 was previously validated in NCAM1 null tissue (Table S1). Furthermore, western blot performed with HRP-conjugated anti-goat, anti-mouse, and anti-rabbit secondary antibodies without primary antibodies revealed nonspecific bands (Fig. S2*B*, open arrowhead). However, none of these bands corresponded to the ∼75 kDa NCAM1-βCTF. Similar to NCAM1, full-length NCAM2-TM was detected by anti-C-terminal NCAM2 (GTX89311) antibody, while the GPI-anchored NCAM2 (NCAM2-GPI) isoform was not detected owing to the absence of the C-terminal cytoplasmic domain (Fig. 1, *A* and *G*, arrow). In contrast to NCAM1, we did not detect the BACE1-specific NCAM2-CTF (NCAM2-βCTF) in the membrane fraction of BACE1+/+ OB even after a longer exposure of the same blot. Lack of detection is most likely owing to the level of NCAM2-βCTF being below the detection limit of western blot, to its rapid degradation, or to its further proteolytic processing.

Based on our finding that NCAM1 and NCAM2 are BACE1 substrates in the OB, NCAM1 and NCAM2 are expected to be colocalized with BACE1 in this brain region. Thus, we performed immunostaining of BACE1+/+ OB sections with anti-BACE1, anti-NCAM1, and anti-NCAM2 antibodies. As previously reported (19, 50), we found that NCAM1 and BACE1 immunostaining significantly overlapped in glomeruli. Similar to NCAM1, there was a significant overlap between NCAM2 and BACE1 in glomeruli (Fig. 1*H*). To ensure the specificity of immunostaining, we used antibodies validated on BACE1, NCAM1, and NCAM2 null tissues (anti-BACE1 antibody 3D5 (51) and Table S1). Furthermore, immunostaining with secondary antibodies in the absence of primary antibodies showed negative or minor nonspecific immunoreactivity after increasing the laser gain (Fig. S3). It has been shown that BACE1 protein is highly enriched in the axonal presynaptic terminal of olfactory sensory neurons (OSNs) in glomeruli (52). Therefore, these data suggest that NCAM1 and NCAM2 may be processed by BACE1 within the axonal terminal of OSNs in the olfactory bulb glomerulus.

### NCAM2 is cleaved by metalloproteinases or BACE1, followed by γ-secretase in HEK cells

**Figure 2:**
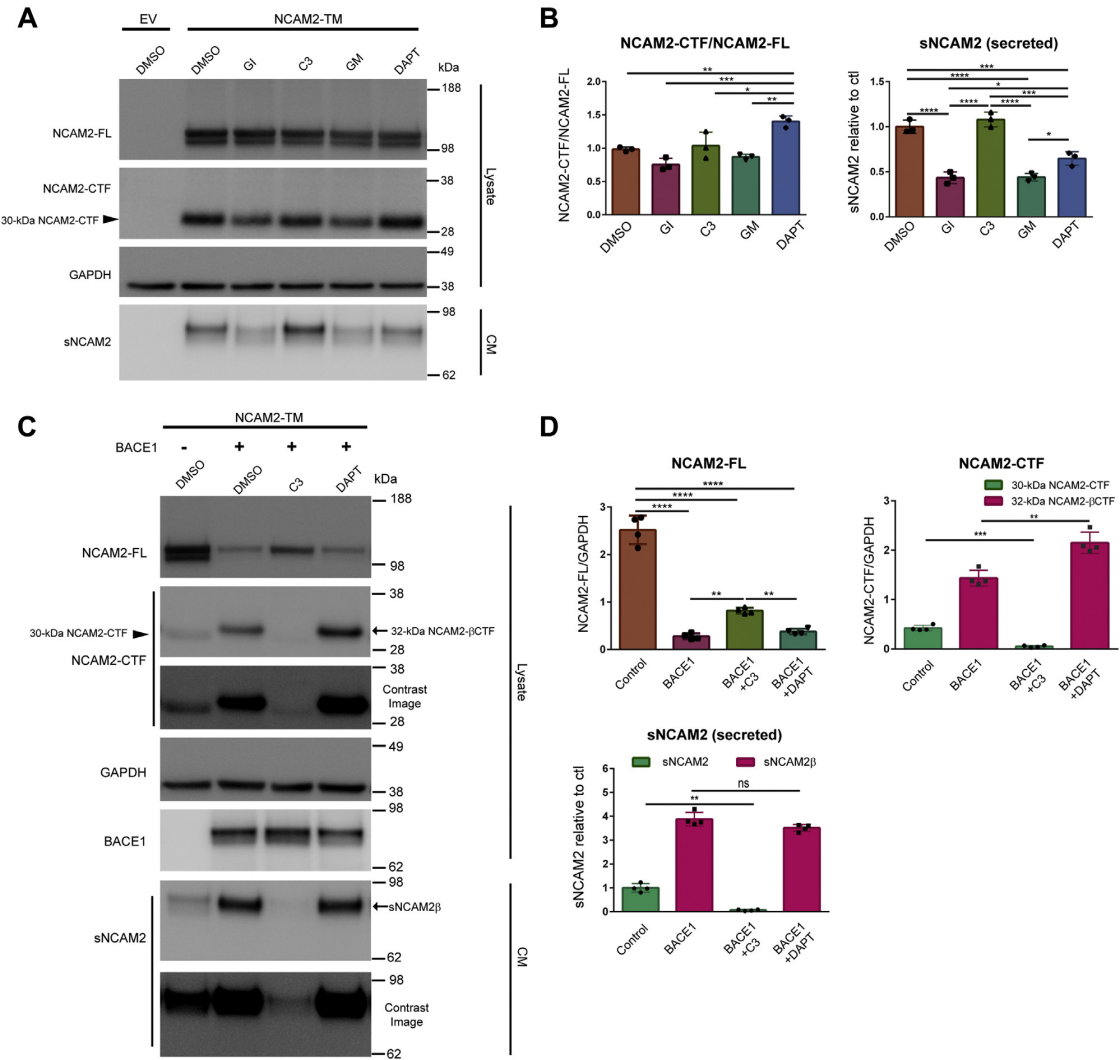
**NCAM2 is cleaved by metalloproteinases or BACE1, followed by γ-secretase in HEK cells.** HEK cells were transfected with NCAM2-Myc-DDK expression vector (transmembrane NCAM2 isoform) or empty vector (EV) as a control and then treated with DMSO only, GI254023X (GI; selective ADAM10 inhibitor, 5 μM in DMSO), C3 (β-secretase inhibitor IV, 10 μM in DMSO), GM6001 (GM; broad spectrum of MMPs inhibitor, 2.5 μM in DMSO), and DAPT (γ-secretase inhibitor, 10 μM, in DMSO) for 24 h as indicated. Total DNA concentration was kept constant at 1.5 μg with empty vector. *A*, representative immunoblot of cell lysates (Lysate) and conditioned media (CM) using anti-Myc (2272), anti-NCAM2 (sc-136328), anti-BACE1 (D10E5), and anti-GAPDH (MAB374) antibodies. Ectopically expressed full-length NCAM2 (NCAM2-FL) in HEK cells undergoes proteolysis with the generation of a C-terminal fragment (NCAM2-CTF) in cell lysates and secreted soluble fragments (sNCAM2) in conditioned media. GI and GM treatments produced a significant reduction of sNCAM2 levels indicating that NCAM2 is cleaved by ADAM10 and MMPs. DAPT treatment results in the accumulation of NCAM2-CTF in cell lysates indicating that NCAM2-CTF is further processed by γ-secretase. *C*, HEK cells were cotransfected with NCAM2 and BACE1 or EV as control and then treated with solvent only (DMSO), C3, or DAPT for 24 h. The ectopic expression of BACE1 produces BACE1-specific NCAM2-CTF (NCAM2-βCTF) and soluble NCAM2 fragment (sNCAM2β), and their production is completely inhibited by C3 treatment. Note that a different protein ladder was used in this figure compared to Figure 1. *B* and *D*, graphs represent densitometric quantification of NCAM2-CTF/NCAM2-FL, NCAM2-FL/GAPDH, NCAM2-CTF/GAPDH ratio in lysates, and secreted soluble NCAM2 (sNCAM2 or sNCAM2β) in CM. One-way ANOVA with Tukey's multiple comparison test was applied. ∗*p* < 0.05, ∗∗<0.01, ∗∗∗*p* < 0.001, ∗∗∗∗*p* < 0.0001, *A* and *B*, n = 3; *C* and *D*, n = 4. Full-length versions of the western blots in *C* are shown in Figure S4A. ns, not significant.

NCAM2 undergoes proteolysis even in the absence of BACE1 (Fig. 1*D*), thus we set out to determine the other proteases responsible for its processing. Previous studies reported that NCAM1 is cleaved by a number of metalloproteinases (46, 47, 48). Given that NCAM2 has the same overall structure as NCAM1, we next determined whether metalloproteases cleave NCAM2 as well. Proteolysis of NCAM2 was analyzed in a cell culture system. First, an expression vector containing C-terminally Myc-DDK-tagged transmembrane isoform of mouse NCAM2 (NCAM2-TM) or an empty vector, as a negative control, was transfected into HEK cells. Then, the activity of a specific protease or a family of proteases was inhibited for 24 h by treatment of the cell cultures with the following drugs: GI254023X (GI; selective ADAM10 inhibitor, 5 μM), C3 (β-secretase inhibitor IV, 10 μM), GM6001 (GM; broad spectrum of MMPs inhibitor, 2.5 μM), or DAPT (γ-secretase inhibitor, 10 μM) (Fig. 2, *A* and *B*). Given that the inhibitors were dissolved in DMSO, a set of cultures was treated with DMSO only as a negative control. Western blot analysis revealed that NCAM2 proteolysis produced a C-terminal fragment of NCAM2 with a size of ∼30 kDa (30-kDa NCAM2-CTF) in the cell lysates and released soluble N-terminal fragments of NCAM2 (sNCAM2) in the conditioned media (CM). NCAM2 processing was assessed by quantifying the ratio of NCAM2-CTF to NCAM2-FL in cell lysates. sNCAM2 levels were measured in a volume of CM adjusted to the protein concentration of cell lysates. Treatment of the cells with either GI or GM resulted in the slight reduction of the NCAM2-CTF/NCAM2-FL ratio in cell lysates and a significant decrease of sNCAM2 in the CM. In contrast, the inhibition of BACE (C3) did not produce any change in the NCAM2-CTF/NCAM2-FL ratio and sNCAM2 levels. These data indicate that NCAM2 is primarily cleaved by ADAM10 and MMPs in HEK cells (Fig. 2, *A* and *B*), most likely owing to low expression of BACE1 in these cells. Importantly, the inhibition of γ-secretase (DAPT) increased the NCAM2-CTF/NCAM2-FL ratio in cell lysates, indicating that this fragment is further cleaved by γ-secretase. We also observed decreased levels of sNCAM2 in the CM from DAPT-treated HEK cells. A possible explanation for this finding is that γ-secretase cleaves not only the NCAM2-CTF but also the NCAM2-FL independently of prior processing by other enzymes (ADAM10 and MMPs). Similarly, a recent study showed that APP-like protein 1 (APLP1), a well-known BACE1 substrate, is cleaved by γ-secretase without previous ectodomain shedding by α- or β-secretase (53).

Next, β-secretase processing of NCAM2 was analyzed following the coexpression of NCAM2-Myc-DDK (NCAM2-TM isoform) and BACE1-GFP in HEK cells (Figs. 2 and S4*A*). Overexpression of BACE1 resulted in the generation of a C-terminal fragment of NCAM2 with the size of ∼32 kDa (32-kDa NCAM2-βCTF) in cell lysates, in contrast to the 30-kDa NCAM2-CTF detected in lysates from cells expressing NCAM2 alone (Fig. 2*C*, lane 1 and lane 2). The 30-kDa NCAM2-CTF was almost undetectable in the lysates of cells coexpressing NCAM2 and BACE1 indicating that NCAM2 processing by ADAM 10 and MMPs is decreased when BACE1 is overexpressed. Accordingly, the majority of sNCAM2 in the CM is a BACE1-specific soluble fragment of NCAM2 (sNCAM2β) (Fig. 2*C*, lane 2). The production of these BACE1-specific 32-kDa NCAM2-βCTF and sNCAM2β concurred with a significant decrease of NCAM2-FL in cell lysates (Fig. 2, *C* and *D*). Furthermore, BACE inhibition (C3) completely blocked the generation of 32-kDa NCAM2-βCTF in cell lysates and sNCAM2β in CM, while 30-kDaNCAM2-CTF and sNCAM2, generated by ADAM10 and MMPs processing, were detected as well as a significant increase in NCAM2-FL (Fig. 2*C*, lane 1–3, and Fig. 2*D*). Altogether, these data indicate that NCAM2 is cleaved by BACE1 *in vitro*. Furthermore, the levels of 32-kDa NCAM2-βCTF were significantly increased in cell lysates treated with DAPT without a significant change in sNCAM2β levels, indicating that the 32-kDa NCAM2-βCTF is further cleaved by γ-secretase, similar to the 30-kDa NCAM2-CTF (Fig. 2*C*, lane 2 and lane 4, and Fig. 2*D*).

### NCAM1 is cleaved by metalloproteinases or BACE1, but not sequentially cleaved by γ-secretase in HEK cells

**Figure 3:**
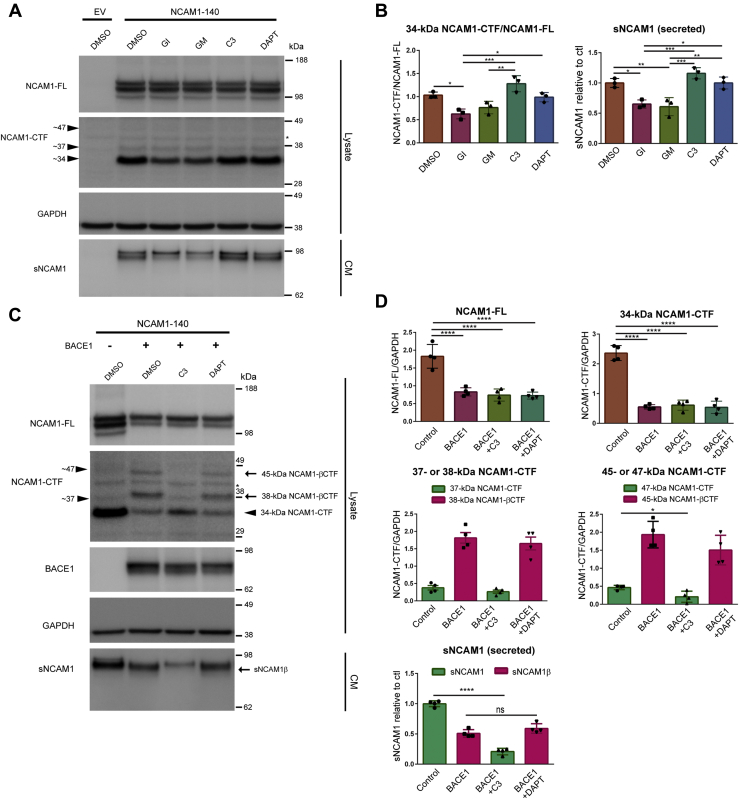
**NCAM1 is cleaved by metalloproteinases or BACE1, but not sequentially cleaved by γ-secretase in HEK cells.** HEK cells were transfected with an NCAM1-Myc-DDK expression vector (NCAM1-140 isoform) or an empty vector (EV) as a control and then treated with DMSO only or indicated inhibitors dissolved in DMSO; GI254023X (GI; selective ADAM10 inhibitor, 5 μM), GM6001 (GM; broad spectrum of MMPs inhibitor, 2.5 μM), C3 (inhibitor IV, 10 μM), and DAPT (γ-secretase inhibitor, 10 μM) for 24 h as indicated. Total DNA concentration was kept constant at 1.5 μg for the empty vector. *A*, representative immunoblot of cell lysates (Lysate) and conditioned media (CM) using anti-Myc (2272), anti-NCAM1 (AF2408), anti-BACE1 (D10E5), and anti-GAPDH (MAB374) antibodies. Ectopically expressed NCAM1 undergoes proteolysis with the production of three C-terminal NCAM1 fragments (NCAM1-CTFs): a major fragment of ∼34 kDa, and two additional fragments of ∼37 kDa, and ∼47 kDa (see *arrowheads*) and secreted soluble fragments (sNCAM1) in conditioned media. The *asterisk* denotes nonspecific bands. GI and GM treatments significantly decreased the levels of NCAM1-CTF (∼34 kDa) and sNCAM1, indicating that NCAM1 is cleaved by ADAM10 and MMPs. *C*, HEK cells were cotransfected with NCAM1 and BACE1 or empty vector as a control and then treated with DMSO, C3, or DAPT for 24 h. Note that the ectopic expression of BACE1 results in cleavage of NCAM1 and the production of BACE1-specific NCAM1-CTF (NCAM1-βCTFs; ∼38 kDa and ∼45 kDa) and soluble NCAM1 (sNCAM1β). However, NCAM1-βCTFs were not accumulated by DAPT treatment, suggesting that NCAM1-βCTFs were not sequentially cleaved by γ-secretase. The *asterisk* denotes nonspecific bands. *B* and *D*, graphs represent densitometric quantification of 34 kDa NCAM1-CTF/NCAM1-FL, NCAM1-FL/GAPDH or NCAM1-CTF/GAPDH ratio in lysates and secreted soluble NCAM1 (sNCAM1) in CM. Note that a different protein ladder was used in this figure compared with Figure 1. One-way ANOVA with Tukey's multiple comparison test was applied. ∗*p* < 0.05, ∗∗ *p* <0.01, ∗∗∗*p* < 0.001, ∗∗∗∗*p* < 0.0001, *A–D*, n = 3. Full-length versions of the western blots in *C* are shown in Figure S4B. ns, not significant.

In order to characterize the proteolysis of NCAM1 in HEK cells, we first transfected an expression vector containing C-terminally Myc-DDK-tagged transmembrane 140 kDa isoform of mouse NCAM1 (NCAM1-140) or empty vector and then treated the cells with protease inhibitors (GI, GM, C3 or DAPT) as described above (Fig. 2, *A* and *B*). Interestingly, proteolysis of ectopically expressed NCAM1 in HEK cells generates three different C-terminal fragments of NCAM1 (a major NCAM1-CTF with a size of ∼34 kDa, and two additional CTFs with a size of ∼37 kDa and ∼47 kDa) (Fig. 3*A* and Fig. S5). Taking in account that the Myc-DDK tag has a weight of ∼3.6 kDa, the major 34-kDa NCAM1-CTF corresponds to a previously described ∼30 kDa NCAM1-CTF (47, 49). These fragments are most likely produced by a number of metalloproteinases (46, 47, 48). NCAM1 processing was assessed by quantifying the ratio of the major NCAM1-CTF of ∼34 kDa (34-kDa NCAM1-CTF) to NCAM1-FL in cell lysates. sNCAM1 levels were measured in a volume of CM adjusted to the protein concentration of the cell lysates. Accordingly, treatment of the cells with either GI or GM resulted in a reduction of the 34-kDa NCAM1-CTF/NCAM1-FL ratio and secreted soluble fragments of NCAM1 (sNCAM1) in cell lysates and in CM, respectively. In contrast, BACE inhibition (C3) did not produce any change of the 34-kDa NCAM1-CTF/NCAM1-FL ratio or sNCAM1 levels (Fig. 3, *A* and *B*). Furthermore, pharmacological inhibition of ADAM10 and metalloproteases prevented the generation of the 34-kDa NCAM1-CTF in agreement with previous studies (47, 49). Similar to NCAM2, these data indicate that NCAM1 is primarily cleaved by ADAM10 and MMPs in HEK cells, most likely owing to low expression of BACE1 in these cells. In contrast to NCAM2, the inhibition of γ-secretase (DAPT) did not produce any change of the 34-kDa NCAM1-CTF/NCAM1-FL ratio in cell lysates and sNCAM1 levels in CM were unchanged, indicating that NCAM1-CTFs are not further cleaved by γ-secretase.

Next, β-secretase processing of NCAM1 was analyzed following the coexpression of NCAM1-Myc-DDK (NCAM1-140 isoform) and BACE1-GFP in HEK cells (Figs. 3 and S4*B*). The ectopic expression of BACE1 with NCAM1 resulted in the generation of NCAM1-CTFs with different molecular weights compared with the ones detected in cell lysates expressing NCAM1 alone. The production of these BACE1-specific C-terminal fragments of NCAM1 (NCAM1-βCTFs) with a size of ∼38 kDa and ∼45 kDa concurred with a decrease of 34-kDa NCAM1-CTF (Figs. 3*C* and S1), indicating that NCAM1 is cleaved by BACE1 at two different sites in its ectodomain *in vitro*. The levels of NCAM1-βCTF with a size of ∼38 kDa (38-kDa NCAM1-βCTF) were relatively higher than the levels of NCAM1-βCTF with a size of ∼45 kDa (45-kDa NCAM1-βCTF). There are two possible explanations for these findings: (1) BACE1 preferentially cleaves NCAM1 at a site near the transmembrane region that generates the 38-kDa NCAM1-βCTF rather than at the site that generates 45-kDa NCAM1-βCTF; and (2) the 45-kDa NCAM1-βCTF is sequentially cleaved by BACE1, resulting in the increased 38-kDa NCAM1-βCTF compared with 45-kDa NCAM1-βCTF. Similarly, previous studies showed that BACE1-derived C99 can be further cleaved at the β′-site by elevated BACE1 activity *in vitro*, resulting in the increased C89 (6, 54). In addition to NCAM1-βCTFs, β-secretase processing of NCAM1 released sNCAM1 in CM with a different molecular weight compared with the one detected in CM of cells expressing NCAM1 only (Fig. 3*C*, lane 1 and 2), indicating that a BACE1-specific soluble fragment of sNCAM1 (sNCAM1β) is produced (Fig. 3*C*, arrow). The production of these BACE1-specific NCAM1-βCTF and sNCAM1β concurred with a significant decrease of NCAM1-FL in cell lysates (Fig. 3, *C* and *D*). Similar to NCAM2, BACE inhibition (C3) completely impaired the generation of NCAM1-βCTFs in cell lysates and sNCAM1β in CM, while NCAM1-CTFs and sNCAM1, generated by ADAM10 and MMPs processing, were observed. Altogether, these data indicate that NCAM1 is cleaved by BACE1 *in vitro*. In contrast to NCAM2-CTFs, DAPT treatment did not result in the accumulation of NCAM1-βCTFs (38-kDa and 45-kDa NCAM1-βCTF) and 34-kDa NCAM1-CTF in cell lysates, indicating that cleaved C-terminal fragments of NCAM1 are not sequentially processed by γ-secretase. The NCAM1-βCTF at ∼75 kDa observed in the OB (Fig. 1, *E* and *F* and Fig. S2*A*, arrowhead) was not observed in HEK cells overexpressing the NCAM1-140 isoform. A possible explanation of this different result is that the 75-kDa NCAM1-βCTF is generated by BACE1 cleavage of the NCAM1-180 isoform *in vivo*.

### Identification and validation of the BACE1 cleavage site in NCAM2 and NCAM1

**Figure 4:**
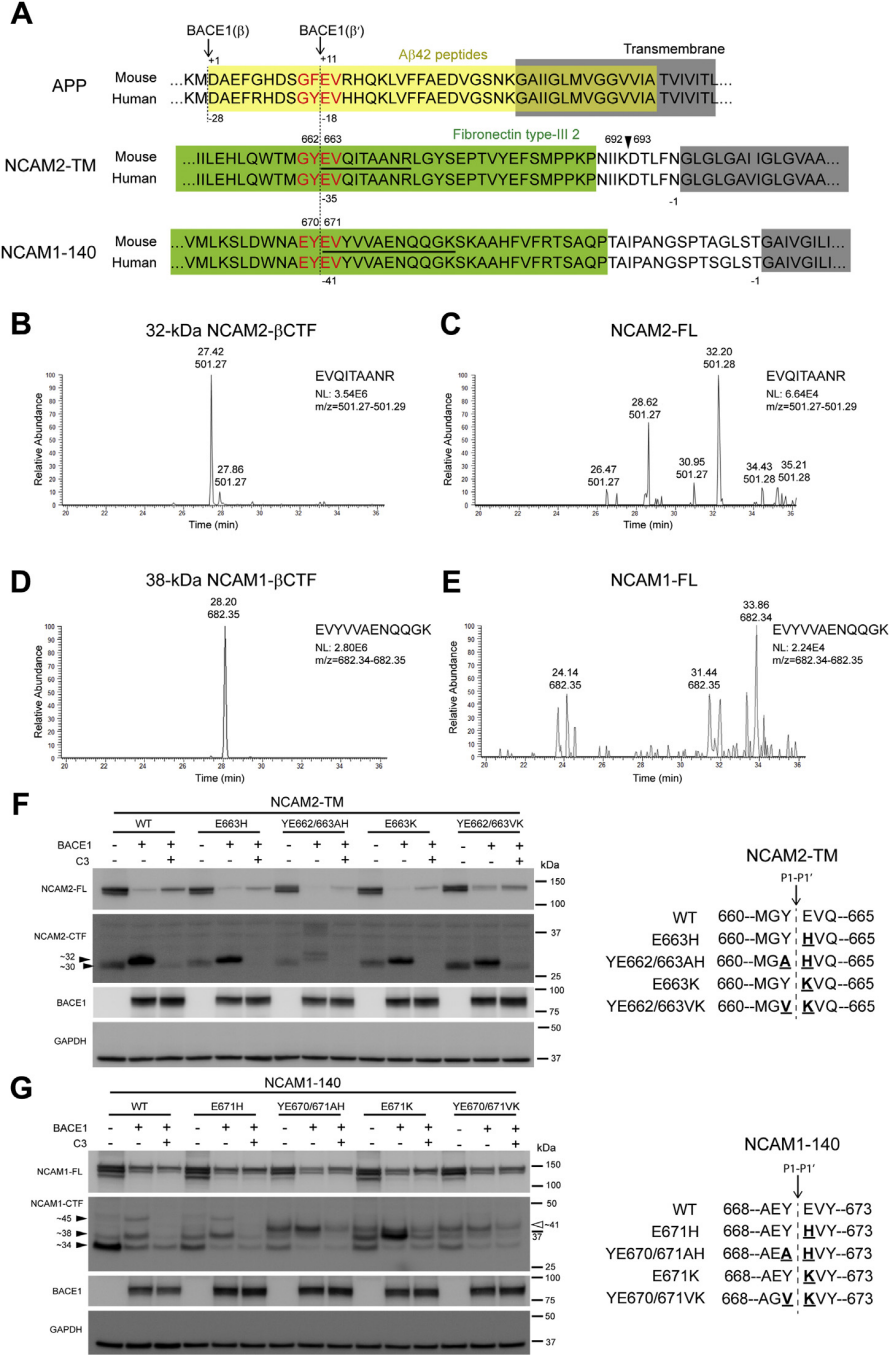
**Identification and validation of the BACE1 cleavage site in NCAM2 and NCAM1.** HEK cells were cotransfected with BACE1 and transmembrane NCAM2 (NCAM2-TM) or NCAM1-140. NCAM2-FL and NCAM2-βCTFs; or NCAM1-FL and NCAM1-βCTF were immunoprecipitated in cell lysates using anti-Myc (911B) antibody with agarose beads. After electrophoresis of immunoprecipitated samples, Coomassie stained bands of NCAM2-βCTF (∼32 kDa, in Fig. 2*C*) and NCAM1-βCTF (∼38 kDa, in Fig. 3*C*) were cut from the gel and sequenced by microcapillary LC/MS/MS to identify the BACE1 cleavage site. NCAM2-FL and NCAM1-FL were also sequenced as a control. *A*, comparison of BACE1 cleavage sites on mouse and human APP, NCAM2, and NCAM1. BACE1 cleaves APP at Asp1 within Aβ peptides (*yellow box*) and also cleaves APP at Glu11 (G^680^Y^681^ ↓ E^682^V^683^) within Aβ peptides, which are located 28 and 18 residues distant from the transmembrane region (*gray box*), respectively. APP numbering is based on the 770 amino acid isoform. NCAM2 and NCAM1 numbering is that of the mouse NCAM2-TM and mouse NCAM1-180 amino acid isoform, respectively. The ↓ symbol denotes the scissile (cleavage) bond. BACE1 cleavage sites of NCAM2 (G^661^Y^662^ ↓ E^663^V^664^) and NCAM1 (E^669^Y^670^ ↓ E^671^V^672^) in the second fibronectin type-III repeat domain (*green box*, see FN2 in Fig. 1*C*) are located 35 and 41 residues distant from the transmembrane region (*gray box*), respectively. Notably, these cleavage sites are identical or similar to the secondary BACE1 cleavage site (β′) of human APP site (GY↓EV). The cleavage sites of NCAM2 at Asp 693 are indicated by the arrowhead. *B* and *C*, ion chromatogram of the peptide peak (EVQITAANR; m/z = 501.27–501.29) from the stained bands (32-kDa NCAM2-βCTF and NCAM2-FL as control). *D* and *E*, ion chromatogram of the peptide peak (EVQITAANR; m/z = 682.34–682.35) from the stained bands (38-kDa NCAM1-βCTF and NCAM1-FL as control). *F* and *G*, BACE1 cleavage site is denoted by a down arrow depicting the scissile bond (P1-P1′). All of the site-directed mutageneses in P1-P1′ amino acids are denoted. HEK cells were cotransfected with BACE1 and wild-type (WT) NCAM or mutant NCAM and then conditioned for 24 h with solvent only (DMSO) or BACE inhibitor (C3, 10 μM) dissolved in DMSO. Total DNA concentration was kept constant at 1.5 μg with empty vector. Cell lysates were analyzed by immunoblot to assess the BACE1 processing of mutant NCAM2 (E663H, YE662/663AH, E663K, YE662/663VK) (*F*) or NCAM1 (E671H, YE670/671AH, E671K, YE670/671VK) (*G*) compared with WT. When BACE1 is overexpressed, only NCAM2-YE662/663AH prohibits the generation of BACE1-specific 32-kDa NCAM2-βCTF (*arrowhead*). Instead, additional bands are produced, but these bands are abolished by BACE inhibition (C3). These data indicate that BACE1 cleaves NCAM2 between Y^662^ and E^663^. NCAM1-E671H mutation did not prevent the generation of NCAM1-βCTFs at ∼38 kDa and ∼45 kDa (*arrowhead*). The ectopic expression of NCAM1-YE670/671AH, NCAM1-E671K, and NCAM1-YE670/671VK generates additional C-terminal fragments of NCAM1 at ∼41 kDa (41-kDa NCAM1-CTF) compared with NCAM1-WT even in the absence of BACE1 expression (*open arrowhead*). The coexpression of NCAM1-E671K with BACE1 resulted in increased 38-kDa NCAM1-βCTF levels, concurrently with reduced 45-kDa NCAM1-βCTF levels, most likely by favoring the proteolysis of NCAM1 at Glu 671 by BACE1. In contrast, only double mutations at the P1-P1′ site of NCAM1 (YE670/671AH and YE670/671VK) abolished the generation of BACE1-specfic 38-kDa NCAM1-βCTF and 45-kDa NCAM1-βCTF, but generated 41-kDa NCAM1-CTF, which was reduced by BACE inhibition (C3). These data support that BACE1 cleaves NCAM1 between Y^670^ and E^671^. *F* and *G*, n = 3.

We next set out to identify the BACE1 cleavage site of NCAM2 using mass spectrometry. First, we cotransfected NCAM2-Myc-DDK (transmembrane isoform of mouse NCAM2; NCAM2-TM) and BACE1-GFP vectors into HEK cells. Then, we immunoprecipitated NCAM2-βCTF and NCAM2-FL from cell lysates using an anti-Myc antibody. Immunoprecipitated samples were separated by electrophoresis and then Coomassie-stained bands corresponding to 32-kDa NCAM2-βCTF and NCAM2-FL were excised, digested with trypsin, and analyzed by microcapillary LC/MS/MS at the Taplin Mass Spectrometry Facility (Harvard Medical School). N-terminal protein was determined by matching the peptide sequence from trypsin digestion to an NCAM2 protein database (Fig. 4*A*). Peptide analysis revealed that an EVQITAANR peptide was highly enriched in 32-kDa NCAM2-βCTF compared with NCAM2-FL, which was used as a control (Fig. 4*A*, underlined peptides, Fig. 4, *B* and *C*, Fig. S6, and Table S2). These data indicate that BACE1 cleaves NCAM2 at Glu 663 (G^661^Y^662^ ↓ E^663^V^664^; the ↓ symbol denotes the scissile bond) in the second fibronectin type-III repeat domain. Notably, this cleavage site (GY↓EV) is identical to the secondary BACE1 cleavage site (β′) of human APP site (GY↓EV). The BACE1 cleavage site in NCAM2 is located 35 residues distant from the transmembrane region, and it is conserved between mouse and human (Fig. 4*A*).

Similar to NCAM2-βCTF, Coomassie stained bands corresponding to 38-kDa NCAM1-βCTF, 45-kDa NCAM1-βCTF, and NCAM1-FL were excised, digested with trypsin, and analyzed by microcapillary LC/MS/MS. Peptide analysis revealed that an EVYVVAENQQGK peptide was highly enriched in the 38-kDa NCAM1-βCTF compared with NCAM1-FL (Fig. 4*A*, underlined peptides, Fig. 4, *D* and *E*, Fig. S7, and Table S2). These data indicate that BACE1 cleaves NCAM1 at Glu 671 (E^669^Y^670^ ↓ E^671^V^672^) in the second fibronectin type-III repeat domain. Notably, this cleavage site (EY↓EV) is similar to the secondary BACE1 cleavage site (β′) of human APP site (GY↓EV) as well as the BACE1 cleavage site of NCAM2 (GY↓EV). The BACE1 cleavage site in NCAM1 is located 41 residues distant from the transmembrane region, and it is conserved between mouse and human (Fig. 4*A*). However, N-terminal peptide analysis failed to identify the other BACE1 cleavage site of NCAM1 (45-kDa NCAM1-βCTF), most likely due to the lower amount of this fragment relative to 38-kDa NCAM1-βCTF (Fig. S5).

Next, in order to validate the BACE1 cleavage site of NCAM2, we introduced single or double mutations at the P1-P1′ sites of putative BACE1 cleavage site in the NCAM2-TM sequence (E663H, YE662/663AH, E663K, and E662/663K), according to the subsite specificity of BACE1 (55). Wild-type (WT) or mutant NCAM2 expression vectors were cotransfected with BACE1 or empty vector in HEK cells to test the extent to which these mutants prevent the BACE1 cleavage of NCAM2 compared to WT (Fig. 4*F*). Only the introduction of YE662/663AH double mutation prevented the generation of the 32-kDa NCAM2-βCTF. However, several new additional C-terminal fragments of NCAM2 were produced in the presence of this double mutation. Since these additional fragments are only produced when ectopic BACE1 is expressed, YE662/663AH mutations might affect the secondary and/or tertiary structure of NCAM2, favoring cleavage of NCAM2 by BACE1 at alternative/cryptic sites (56). Accordingly, the generation of these fragments is reduced by pharmacological BACE inhibition (C3). These results further support the LC/MS/MS data showing that NCAM2 is cleaved at Glu 663 (between Y^662^ and E^663^) by BACE1. Leshchyns'ka *et al*. (42) showed that levels of extracellular secreted fragment of NCAM2 were increased in AD brains and that this cleavage was reduced by replacing aspartic acid at 693 with alanine. Thus, we also tested whether BACE1 cleaves NCAM2 at Asp 693 by replacing aspartic acid with alanine (D693A), lysine (D693K), or histidine (D693H). However, none of these NCAM2 mutations impaired the production of 32-kDa NCAM2-βCTF in cell lysates or sNCAM2β in the CM (Fig. S8). These results indicate that BACE1 does not cleave NCAM2 at Asp 693 (between K^692^ and D^693^) *in vitro* (Fig. 4*A*, arrowhead).

Next, we performed site-directed mutagenesis at the P1′ or P1-P1′ sites of the putative BACE1 cleavage site of NCAM1 (E671H, YE670/671AH, E671K, and YE670/671VK). Similar to NCAM2, WT or mutant NCAM1 expression vectors were cotransfected with BACE1 or empty vector in HEK cells to assess the extent to which these mutations prevent the generation of the 38-kDa NCAM1-βCTF. Substitution of glutamic acid at 671 with histidine (E671H) did not prevent the generation of 38-kDa NCAM1-βCTFs (Fig. 4*G*, arrowhead). The ectopic expression of NCAM1-YE670/671AH, NCAM1-E671K, and NCAM1-YE670/671VK resulted in the production of additional cleaved C-terminal fragments of NCAM1 with a size of ∼41 kDa (41-kDa NCAM1-CTF) compared with NCAM1-WT even in the absence of BACE1 expression (Fig. 4*G*, open arrowhead). Similarly, additional cleavage fragments were observed when the ADAM10 cleavage site of NCAM1 was mutated. Furthermore, the mutagenesis of L1, another BACE1 substrate, at the BACE1 cleavage site results in the generation of new fragments (18, 47). This could be explained by these mutations altering the secondary and/or tertiary structure of NCAM1, thus favoring the cleavage of NCAM1 by metalloproteases or other unknown enzymes at additional sites. The coexpression of NCAM1-E671K with BACE1 resulted in increased 38-kDa NCAM1-βCTF levels, concurrently with reduced 45-kDa NCAM1-βCTF levels, most likely by favoring the proteolysis of NCAM1 at Glu 671 by BACE1. Accordingly, BACE inhibition prevented the production of the 38-kDa NCAM1-βCTF. Interestingly, double mutations at the P1-P1′ sites of NCAM1 (YE670/671AH and YE670/671VK) prevented the generation of the 38-kDa and 45-kDa NCAM1-βCTFs, but generated 41-kDa NCAM1-CTF, which were reduced by BACE inhibition. These data indicate that the YE670/671AH and YE670/671VK double mutations result in the BACE1 cleavage of NCAM1 at a site different from the ones that generate 38-kDa and 45-kDa NCAM1-βCTF. Altogether, these results support the LC/MS/MS data showing that BACE1 cleaves NCAM1 at Glu 671 (between Y^670^ and E^671^) *in vitro*.

### Differential spatiotemporal BACE1 processing of NCAM1 and NCAM2 *in vivo*

NCAM1 germline knockout mice showed size reduction of the OB, deficits in spatial learning (Morris Water Maze) (57), and altered distribution of mossy fibers in the HC (58). NCAM2 knockout mice showed disrupted compartmental organization of axons and dendrites in olfactory glomeruli (59). Since the phenotypes of NCAM1 and NCAM2 knockout mice were observed in the HC and/or OB, NCAM1 and NCAM2 processing by BACE1 was analyzed in the HC and OB.

In the mouse brain, NCAM2 expression is detectable by immunoblot at embryonic day 18 (E18), increases to maximum levels at postnatal day 21 (P21), and remains at a similar level in the adult brain (27), while NCAM1 expression remains relatively unchanged with age (27, 60). These indicate that NCAM1 and NCAM2 have a physiological function in the adult brain as well as the developmental brain. Therefore, we analyzed BACE1 processing of NCAM1 and NCAM2 in the HC and OB of BACE1+/+ and BACE1−/− mice at different ages: postnatal day 10 (P10), 4 months (adult), and 12 months (aged).

**Figure 5:**
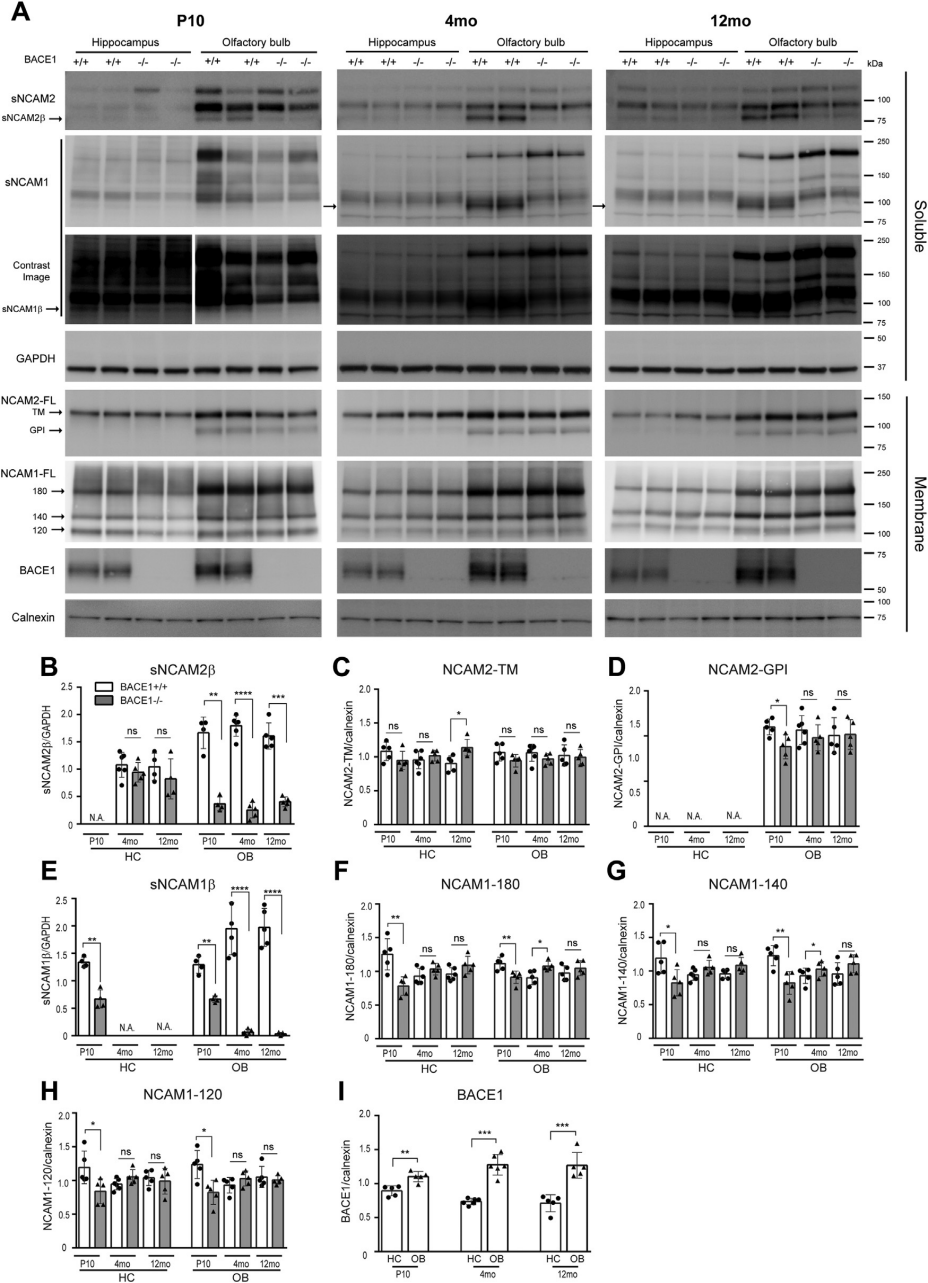
**Differential spatiotemporal BACE1 processing of NCAM1 and NCAM2 *in vivo*.***A*, representative immunoblot of PBS soluble (Soluble) and membrane (Membrane) fraction of the hippocampus (HC) and olfactory bulb (OB) samples from postnatal day 10 (P10), 4-month-old (4 months), and 12-month-old (12 months) BACE1+/+ and BACE1−/− mice. Well-characterized BACE1 substrates *in vivo* are also analyzed in the HC and OB at three different ages using anti-NCAM1 (AF-2408), anti-NCAM2 (sc-136328), BACE1 (D10E5), anti-calnexin (610523), and anti-GAPDH (MAB374) antibodies. After increasing the contrast of the image for sNCAM1, sNCAM1β was detected in HC and OB of BACE1+/+ mice at P10. Transmembrane (TM) and GPI-anchored (GPI) NCAM2 isoforms were observed in the OB membrane fraction, while only NCAM2-TM was detected in HC membrane fraction. Full-length NCAM1 levels (180, 140, and 120) were observed in membrane fraction. Notably, full-length NCAM1 levels are significantly decreased at P10. *B–I*, graphs represent densitometry of soluble protein normalized to GAPDH or densitometry of membrane protein normalized to calnexin. White and gray bars represent BACE1+/+ and BACE1−/− mice, respectively. Unpaired two tailed *t*-test was used for analysis. ∗*p* < 0.05, ∗∗<0.01, ∗∗∗*p* < 0.001, ∗∗∗∗*p* < 0.0001, ns, not significant, N.A.; not applicable, P10 (BACE1+/+; n = 4–5, BACE1−/−; n = 4–5), 4 months (BACE1+/+; n = 5–6, BACE1−/−; n = 5), 12 months (BACE1+/+; n = 4–5, BACE1−/−; n = 4–5). Full-length versions of the western blots (sNCAM2β and sNCAM1β) of 4-months-old mice shown in Figure S1, A and C.

Consistent with our previous findings (Fig. 1*D*), sNCAM2β was almost absent in BACE1−/− OB compared with BACE1+/+ OB at all ages (Fig. 5, *A* and *B*, and Fig. S1, *A* and *B*). However, sNCAM2β was not clearly identified in the HC. Moreover, sNCAM2β and other soluble NCAM2 fragments (sNCAM2) were almost absent in the HC at P10 (Fig. 5*A*), even though the expression of NCAM2 in the HC membrane fraction was clearly detected (Fig. 5*A*). This might suggest that processing of NCAM2 is limited during brain development and NCAM2 is preferentially cleaved by other proteases rather than BACE1 in the HC of adult mice. Transmembrane and GPI-anchored isoforms of NCAM2 (NCAM2-TM and NCAM2-GPI, respectively) were detected in the OB membrane fraction while only NCAM2-TM was detected in the HC membrane fraction. Overall, there was no change in full-length NCAM2 (NCAM2-TM and NCAM2-GPI) levels in the HC and OB between BACE1+/+ and BACE1−/− mice at all ages, with the exception of decreased levels of NCAM2-GPI at P10 BACE1−/− OB and increased levels of NCAM2-TM in 12-month-old BACE1−/− HC (Fig. 5, *C* and *D*). A significant decrease in sNCAM2β levels in BACE1−/− OB was not associated with a detectable increase in full-length NCAM2 levels. A possible explanation for this finding is that NCAM2-FL is processed by compensatory activation of other enzymes including ADAM10 or MMPs in BACE1−/− OB.

Similar to sNCAM2β, sNCAM1β levels were almost absent in the OB from BACE1−/− mice relative to BACE1+/+ in adult and aged mice (Fig. 5*A*, arrow, and Figs. 5*E* and S1*C*). We also found that levels of sNCAM1β are significantly decreased in the OB of BACE1−/− mice relative to BACE1+/+ mice at P10. However, at P10 sNCAM1β was detected as a weak band that could be distinguished from other sNCAM1 only after a longer SDS-polyacrylamide gel electrophoresis separation and by increasing the contrast of the image (Fig. 5*A*). A significant decrease in sNCAM1β levels of BACE1−/− OB was associated with an increase in full-length NCAM1 levels (NCAM1-180, +16%, *p* = 0.018; NCAM1-140, +18%, *p* = 0.014; NCAM1-120, no difference) in adult mice, but not in aged mice (Fig. 5, *F*–*H*). Given that mRNA levels of *Adam10* increase with age (61), unchanged NCAM1-FL levels in aged BACE1−/− mice could be most likely explained by increased NCAM1 processing by ADAM10. We also observed a weak band of sNCAM1β in the HC of BACE1+/+ mice at P10 after increasing the contrast of the image, and it was significantly reduced in BACE1−/− mice (Fig. 5, *A* and *E*). However, this weak band of sNCAM1β was not detected in the HC of adult and aged mice (Fig. 5, *A* and *E*). Accordingly, levels of full-length NCAM1 in BACE1+/+ mice were comparable with the levels of full-length NCAM1 in BACE1−/− mice of adult and aged mice. Unexpectedly, at P10 we observed a significant decrease in NCAM1-FL levels in both HC (NCAM1-180, −60%, *p* = 0.0068; NCAM1-140, −44%, *p* = 0.027; NCAM1-120, −42%, *p* = 0.003) and OB (NCAM1-180, −22%, *p* = 0.009; NCAM1-140; −49%, *p* = 0.003; NCAM1-120 *p* = 0.01) of BACE1−/− mice compared with BACE1+/+ mice (Fig. 5, *A* and *F–H*). NCAM1 is posttranslationally modified by polysialic acid, and this PSA-NCAM1 is predominantly present during embryonic development (27, 34). A recent study showed that PSA modification could mask the epitopes of NCAM1 recognized by anti-N-terminal NCAM1 antibody (62). Therefore, three bands of full-length NCAM1 (NCAM1-FL at ∼120 Da, ∼140 Da, and ∼180 kDa), detected by anti-N-terminal NCAM1 antibody (AF2408), most likely represent nonpolysialylated NCAM1 (non-PSA-NCAM1), suggesting that levels of non-PSA-NCAM1 were significantly decreased in BACE1-deficient mice at P10.

Taken together, these data indicate that NCAM1 and NCAM2 proteins are mainly processed by BACE1 in OB at all ages. However, sNCAM1β was detected in HC only at P10. Interestingly, levels of NCAM1 and NCAM2 in the OB were higher than those in the HC at all ages. Similarly, levels of BACE1 in the OB were higher than those in the HC at all ages (Fig. 5, *A* and *I*). These results might explain why BACE1 processing of NCAM1 and NCAM2 was more evident in the OB than in the HC.

Having established that NCAM1 and NCAM2 are processed differentially by BACE1 in HC and OB in an age-dependent manner, we next analyzed three major *in vivo* validated BACE1 substrates: SEZ6 (24), APP (63), and CHL1 (18, 19) in BACE1+/+ and BACE1−/− HC and OB of P10, adult, and aged mice.

**Figure 6:**
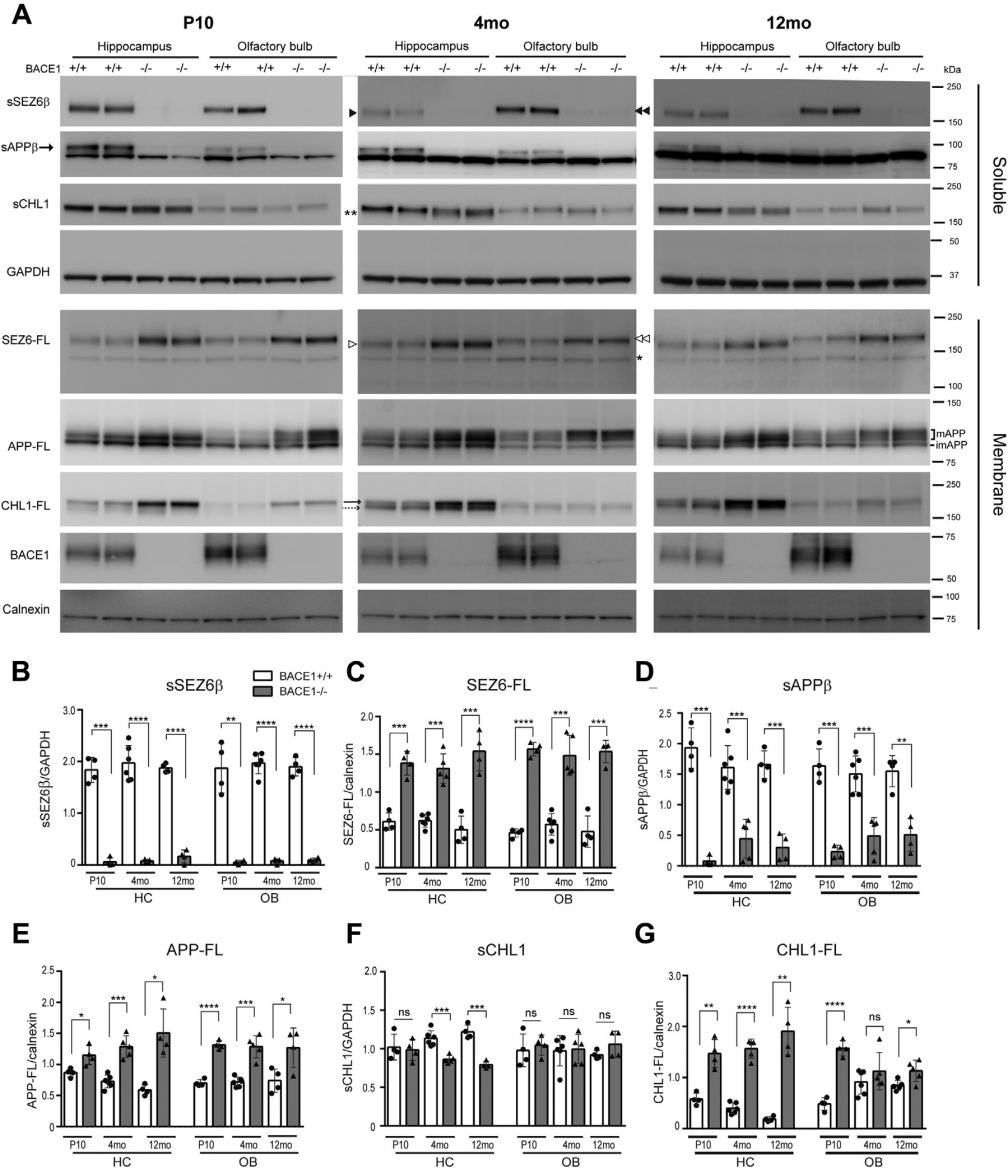
**BACE1 proteolysis of SEZ6, APP, and CHL1 in the hippocampus and olfactory bulb with age.***A*, representative immunoblot of PBS soluble (Soluble) and membrane (Membrane) fractions of the hippocampus (HC) and olfactory bulb (OB) samples from postnatal day 10 (P10), 4-month-old (4 months), and 12-month-old (12 months) BACE1+/+ and BACE1−/− mice. Well-characterized BACE1 substrates are also analyzed in the hippocampus and olfactory bulb at three different ages using anti-SEZ6 (14E5; kindly provided by Dr S. Lichtenthaler), anti-CHL1 (AF2147), anti-sAPPβ (BAWT; kindly provided by Dr S. Lichtenthaler), anti-APP (C1/6.1), anti-NCAM1 (AF-2408), anti-NCAM2 (sc-136328), anti-BACE1 (D10E5), anti-GAPDH (MAB374), and anti-calnexin (610523) antibodies. The molecular weight of sSEZ6β in HC (∼155 kDa, *arrowhead*) is different from the one in the OB (∼165 kDa, *double arrowheads*) at the age of 4 and 12 months. The weak band at ∼140 kDa (*asterisk*) represents immature SEZ6-FL, while the strong band at 165-kDa (*open arrowhead*) or 175-kDa (*double open arrowheads*) in the OB represents mature SEZ6-FL. The different molecular weight of sSEZ6β or SEZ6-FL between the HC and OB of 4- and 12-month-old mice is most likely due to alternative splicing or posttranslational modifications. In the HC membrane fraction, CHL1-FL was detected at ∼185 kDa at P10, while CHL1-FL is detected as two bands at ∼185 kDa (plain arrow) and ∼175 kDa (dash arrow) in 4- and 12-month-old mice, most likely due to splicing variant or glycosylation. *B–G*, graphs represent densitometry of soluble protein (sSEZ6β, sAPPβ, and sCHL1) normalized to GAPDH or densitometry of membrane protein (mature SEZ6-FL, APP-FL, and 185-kDa CHL1) normalized to calnexin. White and gray bars represent BACE1+/+ and BACE1−/− mice, respectively. Unpaired two tailed *t*-test was used for analysis. ∗*p* < 0.05, ∗∗<0.01, ∗∗∗*p* < 0.001, ∗∗∗∗*p* < 0.0001, ns; not significant, P10 (BACE1+/+; n = 4–5, BACE1−/−; n = 4–5), 4 months (BACE1+/+; n = 5–6, BACE1−/−; n = 5), 12 months (BACE1+/+; n = 4–5, BACE1−/−; n = 4–5).

Consistently with a previous study (24), BACE1-specific soluble N-terminal fragment of SEZ6 at ∼165 kDa (sSEZ6β, double arrowheads) was observed in OB soluble fraction of BACE1+/+ mice, but it was almost absent in samples from BACE1−/− mice at all ages (Fig. 6, *A* and *B*). In the OB membrane fraction, full-length SEZ6 (SEZ6-FL) was detected as a strong band at ∼175 kDa (double open arrowheads) and a weak band at ∼140 kDa (Fig. 6*A*, asterisk) at all ages. Interestingly, the molecular weight of sSEZ6β is lower in the HC, ∼155 kDa, (Fig. 6*A*, arrowhead) than in the OB, ∼165 kDa, (Fig. 6*A*, double arrowheads) of adult and aged mice. Similarly, the molecular weight of SEZ6-FL is lower in the HC, ∼165 kDa, (Fig. 6*A*, open arrowhead) than in the OB, ∼175 kDa, (Fig. 6*A*, double open arrowheads) of adult and aged mice. Alternative splicing or posttranslational modifications occurring in the adult brain are likely responsible for the difference in sSEZ6β and SEZ6-FL molecular weight in the HC and OB. Based on a previous study (24), the weak band at ∼140 kDa (Fig. 6*A*, asterisk) represents immature SEZ6-FL containing only high-mannose sugars, while the strong band at ∼165 kDa or ∼175 kDa represents mature SEZ6-FL containing complex sugars such as N- and O-linked glycans (Fig. 6*A*). A significant increase in SEZ6-FL levels was detected in the HC and OB from BACE1−/− mice compared with BACE1+/+ mice, while no change was observed in ∼140 kDa SEZ6-FL levels (Fig. 6, *A* and *C*), indicating that mature SEZ6-FL is cleaved by BACE1 *in vivo*. These results indicate that SEZ6 is predominantly processed by BACE1, regardless of brain region or age.

We next assessed APP processing. APP exists in mature (mAPP; N- and O-glycosylated) and immature (imAPP; O-glycosylated) forms (Fig. 6*A*) (64). Since only mAPP is processed by BACE1 (65), levels of mAPP were significantly increased in HC and OB membrane fractions from BACE1−/− mice at all ages (Fig. 6, *A* and *E*). BACE1-mediated soluble N-terminal APP fragment (sAPPβ) was detected in the soluble fraction of the HC and OB using an anti-sAPPβ specific antibody (BAWT). Similar to SEZ6, sAPPβ was almost absent in HC and OB soluble fractions from BACE1−/− mice (Fig. 6, *A* and *D*) at all ages. These results indicate that proteolytic cleavage of endogenous APP by BACE1 occurs in the HC and OB at all ages.

Finally, the processing of CHL1 was analyzed. In agreement with previous studies (18, 19, 66), we observed significantly decreased levels of the soluble N-terminal fragment of CHL1 (sCHL1) in BACE1−/− HC compared with BACE1+/+ HC of 4- and 12-month-old mice (Fig. 6*F*). However, levels of sCHL1 were comparable in BACE1+/+ and BACE1−/− HC at P10. Similarly, no differences were observed between levels of sCHL1 of BACE1+/+ OB and those of BACE1−/− OB at all ages. In the HC membrane fraction, one major band of full-length CHL1 (CHL1-FL) was detected at ∼185 kDa at P10, while CHL1-FL is detected as two bands at ∼185 kDa (Fig. 6*A*, plain arrow) and ∼175 kDa (Fig. 6*A*, dash arrow) in adult and aged brains. As previously reported (19, 66), 175-kDa CHL1-FL may be a splicing variant or the result of glycosylation of CHL1 in the HC of adult and aged mice. Notably, levels of 185-kDa CHL1-FL were significantly increased in the HC and OB at all ages, except in the OB at the age of 4 months. As previously described (14, 66), an sCHL1 fragment was detected in the soluble fraction from BACE1−/− mice (Fig. 6*A*, double asterisks). Such soluble fragment is most likely derived by a compensatory increased cleavage of CHL1 by ADAM8 or ADAM10 (67, 68). This may explain why we only detected reduced sCHL1 levels in BACE1−/− HC compared with BACE1+/+ samples from adult and aged mice, although levels of 185 kDa CHL1-FL were increased in the HC and OB at all ages, except in the OB at the age of 4 months. Moreover, the much lower expression of CHL1 in the OB relative to the HC might make it difficult to detect the BACE1 processing of CHL1 in the OB. These results indicate that proteolytic cleavage of CHL1 by BACE1 occurs in the HC and OB at all ages. Taken together, SEZ6, APP, and CHL1 by BACE1 are cleaved by BACE1 in the HC and OB at all ages. In contrast, BACE1 differentially processes NCAM1 and NCAM2 depending on the regions of brain and age.

### Polysialylated NCAM1 levels are increased in BACE1−/− mice at P10

**Figure 7:**
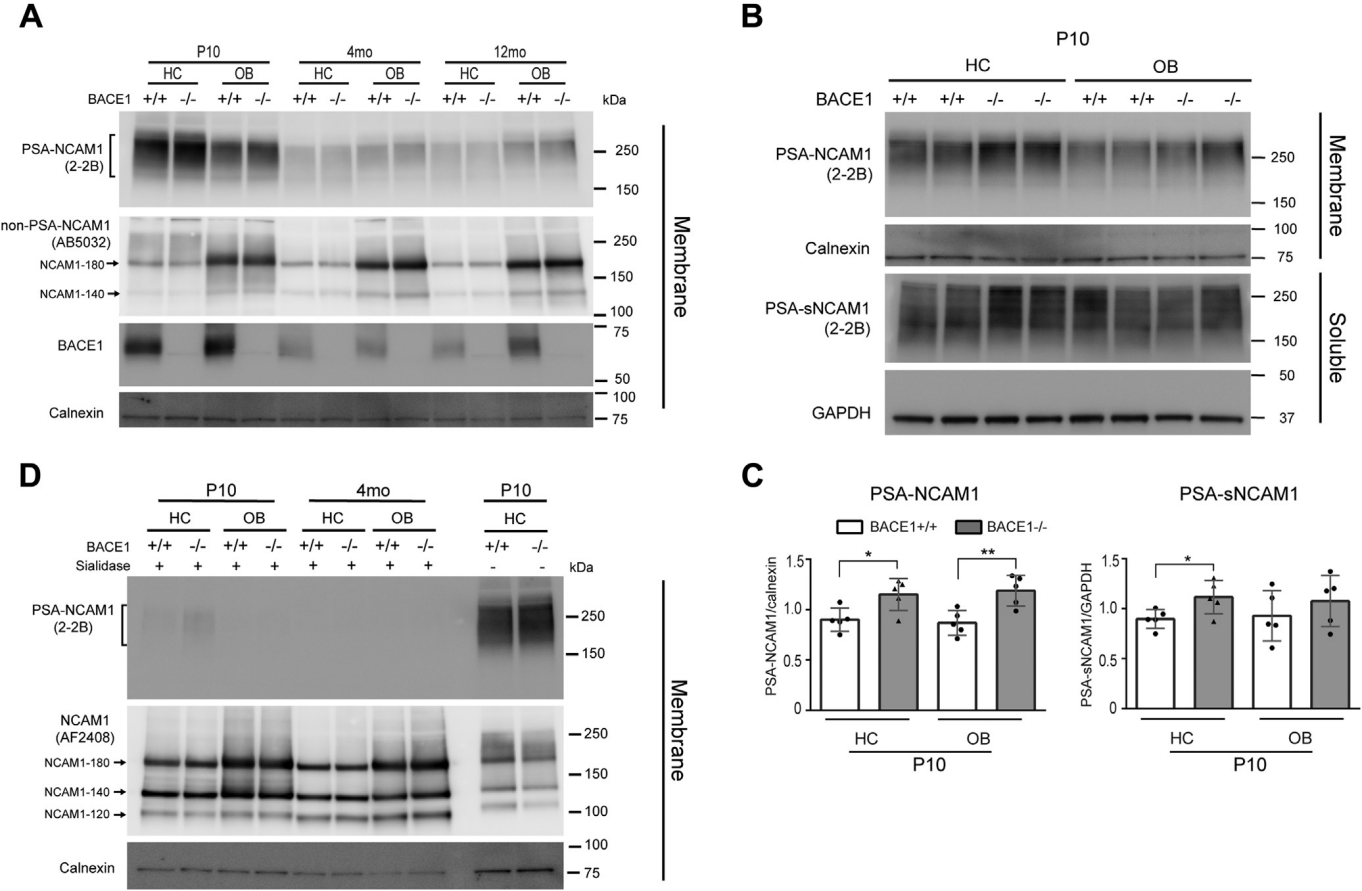
**PSA-NCAM1 levels are increased in BACE1−/− mice at P10.** PBS soluble (Soluble) and membrane (Membrane) fractions of hippocampus (HC) and olfactory bulb (OB) samples from postnatal day 10 (P10), 4-month-old (4 months), and 12-month-old (12 months) BACE1+/+ and BACE1−/− mice were analyzed by immunoblot with anti-PSA-NCAM1 (2-2B), anti-C-terminal NCAM1 (AB5032), anti-N-terminal NCAM1 (AF2408), anti-BACE1 (D10E5), anti-calnexin (610523), and GAPDH (MAB374) antibodies. *A*, PSA-NCAM1 levels were analyzed in HC and OB of BACE1+/+ and BACE1−/− mice during aging. PSA-NCAM1 is predominantly present at P10, especially in the HC. Although PSA modification was significantly decreased with age, total NCAM1 levels remained unchanged. *B*, PSA-NCAM1 levels in HC and OB from BACE1−/− mice were higher than ones in BACE1+/+ mice at P10. Similarly, soluble PSA-NCAM1 (PSA-sNCAM1) levels in the HC from BACE1−/− mice were higher than ones in BACE1+/+ mice at P10. *C*, graphs represent densitometry of PSA-NCAM1 normalized to calnexin and PSA-sNCAM1 normalized to GAPDH. *D*, after treatment of sialidase to remove the sialic acid from NCAM1, total NCAM1 levels were similar between BACE1+/+ and BACE1−/− mice at P10. Unpaired two tailed *t*-test was used for analysis. ∗*p* < 0.05, ∗∗*p* < 0.001, (BACE1+/+; n = 5, BACE1−/−; n = 5).

We showed above that NCAM1 levels, most likely representing non-PSA-NCAM1, were unexpectedly decreased in BACE1−/− mice at P10. Since PSA-NCAM1 is predominantly present during embryonic development, we also measured PSA-NCAM1 levels to determine the levels of total NCAM1 (PSA and non-PSA NCAM1) in BACE1+/+ and BACE1−/− mice. Therefore, we assessed the levels of PSA-NCAM1 in the HC and OB during aging by performing western blot with anti-PSA-NCAM1 (2-2B) antibody. PSA-NCAM1 was detected in the membrane fraction as a broad band with a molecular weight ranging from ∼180 to 300 kDa (Fig. 7*A*). Consistent with previous studies, PSA-NCAM1 was predominantly present in the HC and OB at P10, while PSA-NCAM1 levels were significantly reduced at 4- and 12-months of age (Fig. 7*A*) (27, 34). We found that PSA-NCAM1 levels were higher in the HC compared with the OB while non-PSA-NCAM1 levels, detected by anti-N-terminal (AF2408) or anti-C-terminal (AB5032) NCAM1 antibody, were lower in the HC compared with the OB at P10 (Figs. 5*A* and 6*A*). Therefore, the ratio of PSA-NCAM1 to the non-PSA-NCAM1 was significantly higher in the HC (PSA-NCAM1/non-PSA-NCAM1 = 1.47) compared with the OB (0.66) at P10 (*p* = 0.0003). This result indicates that a higher proportion of NCAM1 is polysialylated in the HC compared with OB at the early developmental stage. Moreover, we observed increased PSA-NCAM1 levels in the HC and OB of BACE1−/− mice compared with BACE1+/+ mice at P10. Levels of soluble PSA-NCAM1 fragments (PSA-sNCAM1) were also increased in the HC, but not in the OB, of BACE1−/− mice compared with BACE1+/+ mice at P10 (Fig. 7, *B* and *C*). In order to determine whether a general increase in levels of NCAM1 was responsible for the increase in PSA-NCAM1 in BACE1−/− samples, the total NCAM1 (PSA-NCAM1 and non-PSA-NCAM1) levels in membrane fraction were assessed after removing PSA modification from NCAM1 with sialidase (P0722) treatment. Western blot analysis with anti-PSA-NCAM1 (2-2B) antibody confirmed the removal of PSA modification compared with the same samples without sialidase treatment (Fig. 7*D*, lanes 1–2 *versus* lanes 9–10). Subsequent western blot analysis with anti-N-terminal NCAM1 (AF2408) antibody revealed that total NCAM1 levels in BACE1+/+ mice were similar to those in BACE1−/− mice at P10 (Fig. 7*D*). These data suggest that PSA modification of NCAM1 is increased in BACE1−/− mice at P10. Moreover, the increased proportion of PSA-NCAM1 in BACE1−/− mice at P10 can explain the observed decrease in the non-PSA-NCAM1 levels, detected by N-terminal antibody (AF2408) (Fig. 5, *A* and *F–H*).

### PSA-NCAM1 is cleaved by BACE1

**Figure 8:**
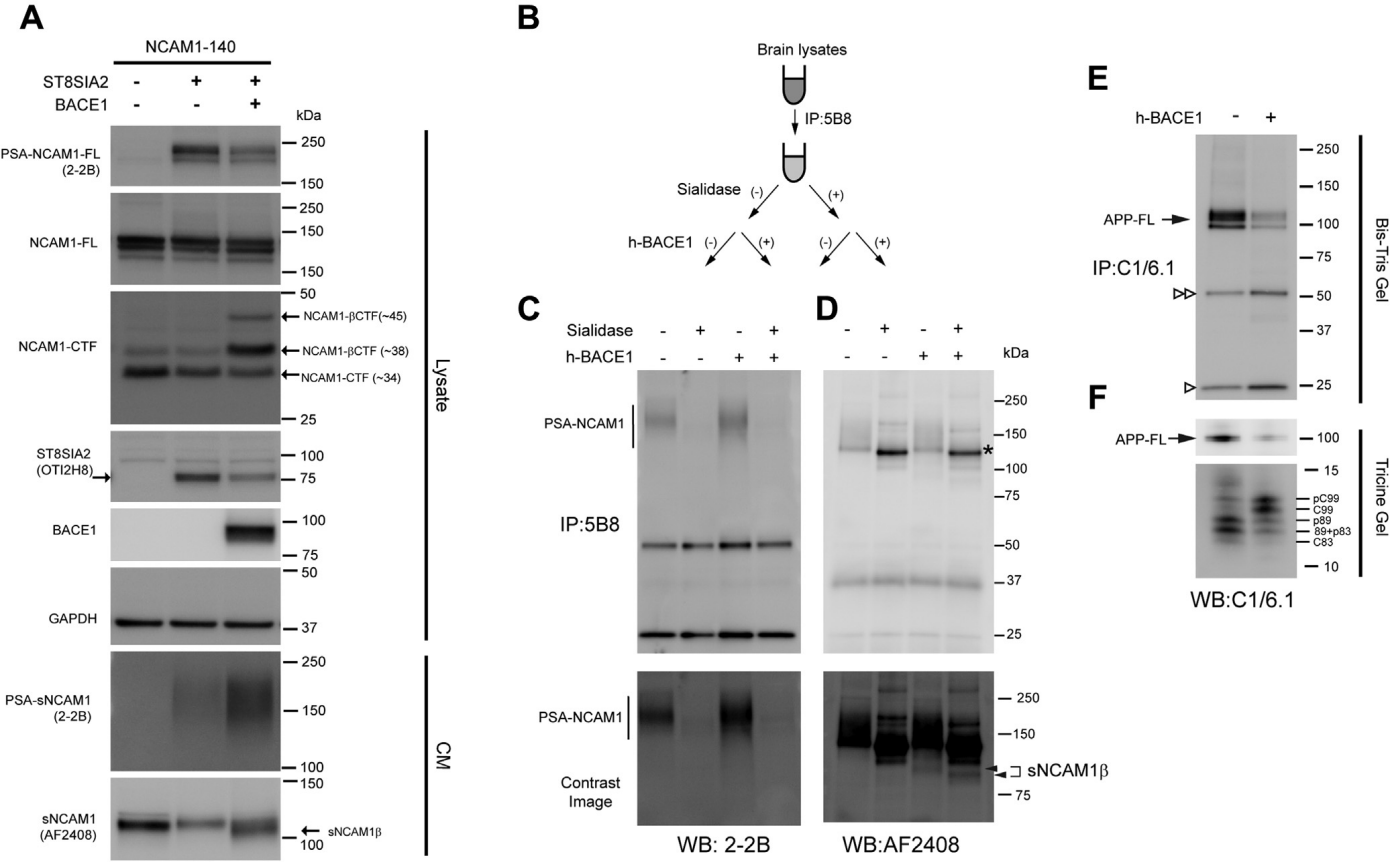
**PSA-NCAM1 is cleaved by BACE1.***A*, HEK cells were transiently transfected with the indicated expression vectors (NCAM1, ST8SIA2, and BACE1) for 48 h. Representative immunoblot of cell lysates (Lysate) and conditioned media (CM) using anti-PSA-NCAM1 (2-2B), anti-Myc (2272), anti-turboGFP (OTI2H8), anti-NCAM1 (AF2408), anti-BACE1 (D10E5), and anti-GAPDH (MAB374) antibodies. ST8SIA2 ectopic expression induces an increase in PSA-NCAM1 levels. Note that the ectopic expression of BACE1 produces BACE1-specific NCAM1-βCTFs (∼38 and ∼45 kDa) in the cell lysates and released sNCAM1β in the CM. Also, secreted soluble PSA-NCAM1 levels were increased. This result indicates BACE1 cleaves PSA-NCAM1 as well as non-PSA-NCAM1 in HEK cells. n = 3. *B–D*, NCAM1 proteins (PSA-NCAM1 and non-PSA-NCAM1) from P10 hemibrain lysates were immunoprecipitated using anti-C-terminal NCAM1 antibody (5B8). Immunoprecipitants were incubated with or without sialidase (P0722) to remove sialic acid modification and were then incubated with or without human recombinant BACE1 (h-BACE1) (*B*). After treatment, samples were directly analyzed by western blot (WB) using anti-PSA-NCAM1 (2-2B) (*C*) or anti-N-terminal-NCAM1 (AF2408) (*D*) antibodies. As expected, PSA-NCAM1 was almost absent in samples incubated with sialidase (lanes 1 and 3). Immunoblot with anti-N-terminal-NCAM1 (AF2408) antibodies revealed the presence of an N-terminal NCAM1 fragment in samples incubated with h-BACE1 independently of sialylation status of NCAM1 (*arrowheads*). *E* and *F*, immunoprecipitated endogenous APP using anti-C-terminal APP antibody (C1/6.1) was incubated with human BACE1 (h-BACE1) as a positive control for the BACE1 enzymatic cleavage experiment. While C99 and C89 represent BACE1-mediated C-terminal APP fragments (APP-βCTFs), C83 is an α-secretase-mediated C-terminal APP fragment (APP-αCTFs). APP-CTFs are present as phosphorylated (pC99, pC89, and pC83) and nonphosphorylated (C99, C89, and C83) forms. h-BACE1 cleaves APP-FL and increased C99 and pC99 levels, concurrently decreased C89/pC89 and C83/pC83 levels. Bands around at 50 kDa (*double open arrowheads*) and 25 kDa (*open arrowheads*) correspond to the heavy and light chain of precipitated primary antibody (C1/6.1), respectively. While a Bis-Tris gel was used to see the full-length APP (*E*), Tris-Tricine gels were used to separate APP-CTFs (*F*).

We next set out to determine whether PSA-NCAM1 as well as non-PSA-NCAM1 could be processed by BACE1. Polysialyltransferase STX, also known as ST8SIA2, is mainly responsible for PSA modification of NCAM1 during the developmental stage (30, 31, 32, 33). First, NCAM1 was ectopically expressed with and without C-terminally turboGFP tagged mouse ST8SIA2 in HEK cells. PSA-NCAM1-FL (∼230 kDa) and PSA-sNCAM1 were detected by western blot with anti-PSA antibody (2-2B) in cell lysates or CM of cells coexpressing NCAM1 and ST8SIA2, but not in cells expressing NCAM1 only (Fig. 8*A* lane 1 *versus* lane 2). Next, BACE1 was cotransfected with ST8SIA2 and NCAM1. Ectopic expression of BACE1 produced NCAM1-βCTF (∼38 and∼45 kDa) in the cell lysates and released sNCAM1β in the CM. Also, we observed an increase in PSA-sNCAM1 levels in the CM, concurrently with a decrease in PSA-NCAM1-FL levels (Fig. 8*A*). These data indicate that BACE1 cleaves PSA-NCAM1 as well as non-PSA-NCAM1 in HEK cells.

In order to confirm our data using mouse brain, NCAM1 proteins from P10 BACE1+/+ brain homogenates were first immunoprecipitated using anti-C-terminal NCAM1 antibody (5B8)-agarose bead complex. Given that PSA-NCAM1 is enriched at P10 (Fig. 7*A*), the NCAM1/5B8-bead complex was incubated in a reaction buffer with or without sialidase to obtain PSA-NCAM1-enriched or non-PSA-NCAM1-enriched antigen–antibody bead complexes, respectively (Fig. S9). After washing with PBS, PSA-NCAM1 or non-PSA-NCAM1-enriched NCAM1/5B8-bead complexes were incubated in a reaction buffer (100 mM sodium acetate, pH 4.5) with or without human recombinant BACE1 (h-BACE1) to compare the BACE1 enzymatic processing of PSA-NCAM1 and non-PSA-NCAM1 (Fig. 8*B*). Because a low pH condition dissociates the antigen–antibody interaction as well as the antibody–bead interaction, each final reaction solution was directly analyzed by western blot without washing with PBS. PSA-NCAM1 was detected with anti-PSA NCAM1 antibody (2-2B) as a broad band with a molecular weight with a range of ∼150–250 kDa (Fig. 8*C*, lanes 1 and 3), but it was almost absent in samples treated with sialidase (Fig. 8*C*, lanes 2 and 4). A NCAM1 fragment was detected in samples with h-BACE1 treatment but not in samples without h-BACE1 treatment, indicating that this represents a BACE1-mediated N-terminal fragment of NCAM1 (sNCAM1β) (Fig. 8*D*, lane 3 and 4, arrowheads). Accordingly, the molecule weight of the sNCAM1β fragment (Fig. 8*D*, lane3, arrowhead) was slightly lower in the sample treated with sialidase compared with the one detected in the sialidase-untreated sample (Fig. 8*D*, lane 4, arrowhead). Furthermore, a slight reduction of non-PSA-NCAM1 levels was observed in a sample incubated with hBACE1 (lane 4) compared with control (lane 2) (Fig. 8*D*, asterisk) owing to BACE1-mediated processing. These data indicate that BACE1 cleaves both non-PSA-NCAM1 and PSA-NCAM1. Immunopurified endogenous APP-FL was used as a positive control for BACE1 enzymatic assay (Fig. 8, *E* and *F*). As expected BACE1 processing of APP reduced the APP-FL levels concurrently with increased BACE1-mediated APP-CTFs including C99 and phosphor C99 (pC99). A possible explanation for the decreased levels of C89/pC89 and C83/pC83 is that recombinant BACE1 cleaves APP-CTFs at noncanonical sites in an *in vitro* assay. Accordingly, Farzan *et al*. observed Aβ (1, 2, 3, 4, 5, 6, 7, 8, 9, 10, 11, 12, 13, 14, 15, 16, 17, 18, 19) and Aβ (1, 2, 3, 4, 5, 6, 7, 8, 9, 10, 11, 12, 13, 14, 15, 16, 17, 18, 19, 20) by matrix-assisted laser desorption/ionization time-of-flight (MALDI-TOF) mass spectrometry after Aβ (1, 2, 3, 4, 5, 6, 7, 8, 9, 10, 11, 12, 13, 14, 15, 16, 17, 18, 19, 20, 21, 22, 23, 24, 25, 26, 27, 28) peptide was incubated with BACE1 at 37 °C for 2 h. This result suggests that BACE1 could cleave at Phe 20 and Ala 21 (Aβ numbering) in an *in vitro* assay (5).

### NCAM1 but not NCAM2 is a BACE1 substrate in hippocampal synaptosomes of adult mice

**Figure 9:**
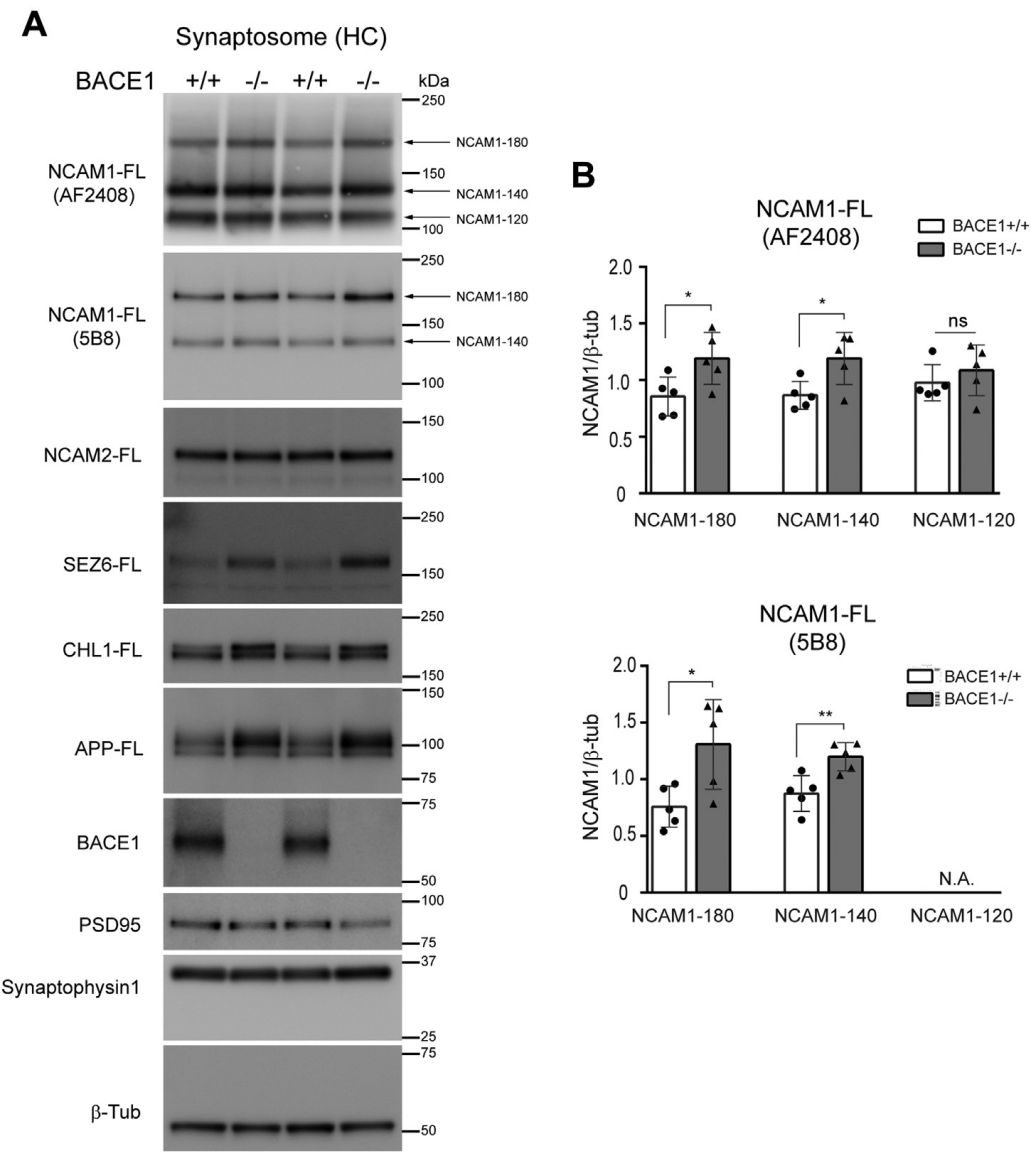
**NCAM1 but not NCAM2 is a BACE1 substrate in hippocampal synaptosomes of adult mice.** Four-month-old BACE1+/+ and BACE1−/− hippocampi were used to isolate the synaptosome using discontinuous sucrose ultracentrifugation. *A*, representative immunoblot of purified hippocampal synaptosomes using anti-NCAM1 (AF2408), anti-NCAM1 (5B8), anti-NCAM2 (sc-136328), anti-SEZ6 (14E5), anti-CHL1 (AF2147), anti-APP (C1/6.1), anti-BACE1 (D10E5), anti-PSD95 (ab18258), anti-synaptophysin1 (101011), and anti-β-tubulin (2146) antibodies. Increased levels of NCAM1-FL (NCAM1-180 and NCAM1-140), CHL1-FL, APP-FL, and SEZ6-FL were detected in hippocampal synaptosomes. *B*, graphs represent densitometry of full-length NCAM1 (NCAM1-180, NCAM1-140, and NCAM1-120) protein with anti-NCAM1 (AF2408) or anti-NCAM1 (5B8) antibody normalized to β-tubulin in synaptosome. Unpaired two tailed *t*-test was used for analysis. ∗*p* < 0.05, ∗∗*p* < 0.001, BACE1+/+; n = 5, BACE1−/−; n = 5. N.A., not applicable; ns, not significant.

NCAMs have been shown to regulate formation, maturation, and maintenance of synapses and are enriched in synaptosomes (37, 42, 69). Since BACE1 is also enriched in synaptosomes (18, 70), we prepared hippocampal synaptosomes from 4-month-old BACE+/+ and BACE1−/− mice using sucrose gradient ultracentrifugation. Presynaptic protein marker, synaptophysin1 and postsynaptic protein marker (PSD95) were used as controls. We found that NCAM1-FL levels, detected by N-terminal (AF2408) or C-terminal NCAM1 (5B8) antibodies, were significantly increased in BACE1−/− hippocampal synaptosomes, compared with BACE1+/+ samples (Fig. 9). However, NCAM2-FL levels were not changed in BACE1−/− hippocampal synaptosome. We also found that SEZ6-FL, CHL1, and APP levels were increased in BACE1−/− samples. This data suggests that BACE1 cleaves NCAM1, but not NCAM2, along with other substrates (CHL1, APP, and SEZ6) in hippocampal synaptosomes. While the total levels of NCAM1 appear to be unaffected in the total extracts of BACE1−/− HC, synaptic NCAM1 levels were increased (Figs. 5 and 9). This could be explained by the proteolysis of adult hippocampal NCAM1 specifically occurring within the synaptosome in adult brains, therefore this might not be accompanied by changes in the levels of NCAM1 in HC total extracts. Similarly, the lack of detection of sNCAM1β, in the soluble fraction, is probably due to the levels of sNCAM1β being below the detection limits of western blot. However, we did not observe significantly increased sNCAM2β and/or synaptic NCAM2 in BACE1−/− HC compared with BACE1+/+ HC. In addition, NCAM2 at Asp 693 was not cleaved by BACE1 *in vitro* (Fig. S8). Thus, NCAM2 does not seem to be cleaved by BACE1 in hippocampal synaptosomes. Therefore, the increased cleavage of NCAM2 observed in AD brains (42) is most likely due to other proteases such as metalloproteinases.

## Discussion

Here, we report that BACE1 differentially processes NCAM1 and NCAM2 depending on the region of brain, subcellular localization, and age *in vivo*. NCAM2 and NCAM1 are highly expressed in OB, thus we first analyzed their processing in the OB of adult mice. We found that both NCAM1 and NCAM2 undergo BACE1-dependent processing as demonstrated by the absence of a specific soluble fragment in the OB of BACE1−/− mice. Accordingly, BACE1, NCAM1, and NCAM2 colocalize in the OB.

Leshchyns'ka *et al*. (42) found that the levels of synaptic NCAM2 were significantly reduced, whereas levels of soluble fragment of NCAM2 were increased in the hippocampus of AD brains compared with controls, indicating an increased proteolysis of NCAM2. However, the proteases responsible for the processing of NCAM2 have not been identified. In contrast, the proteolysis of NCAM1 by ADAM 10 and a number of metalloproteinases (46, 47, 48) has been extensively studied. Given that NCAM2-TM and NCAM1-140 amino acid sequences are ∼44% identical (27), we set out to determine whether NCAM2 is processed by ADAM10 and other metalloproteinases. Similar to NCAM1, we found that overexpressed NCAM2-TM was primarily cleaved by ADAM10 and MMPs and generated a C-terminal fragment of NCAM2 (30-kDa NCAM2-CTF) in HEK cells (Fig. 2*A*). However, BACE1-specific fragments of NCAM2 (32-kDa NCAM2-βCTF and sNCAM2β) and of NCAM1 (38-kD, 45-kDa NCAM1-βCTFs and sNCAM1β) were generated when BACE1 was ectopically expressed. In addition, we found that cleaved NCAM2-βCTF, but not NCAM1-βCTF, was sequentially cleaved by γ-secretase.

Having identified the BACE1-derived of NCAM1 and NCAM2 by BACE1 overexpression and inhibition, we determined BACE1 cleavage sites using microcapillary LC/MS/MS analysis. We found that *in vitro* BACE1 cleaves NCAM2 at Glu 663 (G^661^Y^662^ ↓ E^663^V^664^) in the second fibronectin type-III repeat domain. Similarly, we also found that BACE1 cleaves NCAM1 at Glu 671 (E^669^Y^670^ ↓ E^671^V^672^) in the second fibronectin type-III repeat domain *in vitro*. Notably, these cleavage sites are similar or identical to the secondary BACE1 cleavage site (β′-site) of human APP (GY↓EV) (Fig. 4*A*). BACE1 cleaves APP at Asp1 within Aβ to produce the secreted sAPPβ and membrane-bound C99, whereas it can also cleave APP at Glu11 (G^680^Y^681^ ↓ E^682^V^683^) within Aβ to generate sAPPβ′ and membrane-bound C89 (3, 4, 5, 6). To further confirm these findings, we generated single or double mutations at the P1′ or P1-P1′ sites of NCAM2 and NCAM1 to impair the BACE1-mediated 32-kDa NCAM2-βCTF and 38-kDaNCAM1-βCTF, respectively. Double mutations of NCAM2 (YE662/663AH) and NCAM1 (YE670/671AH and YE670/671VK) prevented BACE1-mediated processing (Fig. 4, *F* and *G*). A previous study showed that Aβ treatment induced cleavage of NCAM2 at aspartic acid 693 (42). Thus, we tested whether BACE1 cleaves NCAM2 at Asp 693 (between K^692^ and D^693^) and found that substitution of D693 to Ala, Lys, or His did not affect the BACE1-mediated processing of NCAM2 *in vitro* (Fig. 4*A*, arrowhead and Fig. S8) suggesting that most likely metalloproteases cleave NCAM2 at D693.

Previous work demonstrated that proteolysis regulates the function of NCAM1 by terminating the function of full-length protein and generation of soluble fragments that further interfere with the function of full-length. NCAM1 is involved in homophilic binding with NCAM1 on the same (cis-interaction by the Ig1-Ig2 domains) and opposing cell surface (trans-interaction; zipper-like interaction between the Ig1-Ig3, Ig2-Ig2, and Ig2-Ig3 domains) (71, 72). These NCAM1-NCAM1 cis- and trans-homophilic interactions are important for neurite outgrowth (71) and promote stabilization of synapses (73). The extracellular domain of NCAM1 also mediates heterophilic binding with other CAMs (L1 and TAG1), extracellular matrix molecules (ECMs) (heparin sulphate proteoglycan (HSPG) and chondroitin sulfate proteoglycans [CSPG]), and receptors (fibroblast growth factor receptor (FGFR1) and GDNF family receptor α [GFRα]) (74, 75). Transgenic mice overexpressing extracellular NCAM1 (NCAM1-EC), including five Ig-like and two fibronectin III domains, have reduced GABAergic inhibitory and excitatory synapses along with altered behavior phenotypes (76). In cortical neuron cultures, soluble NCAM1-EC inhibits NCAM1-dependent neurites branching and outgrowth (77). Therefore, proteolytic processing of NCAM1-FL by metalloproteases or BACE1 could impair the functions of NCAM1-FL. The sNCAM1β fragment produced by proteolysis could acts as a dominant negative by binding to membrane-bound NCAM1-FL or to other NCAM1-binding partners.

In contrast to NCAM1, NCAM2 is known to form homophilic trans-interaction, but not cis-interaction (27). NCAM2 is enriched in synaptosome and is important for maintaining synapses by forming homophilic NCAM2 trans-interaction. Disruption of NCAM2-mediated synaptic adhesion induces disassembly of GluR1-containing glutamatergic synapses in cultured hippocampal neurons (42). Therefore, proteolytic processing of NCAM2 by metalloprotease or BACE1 could impair the function of NCAM2-FL. While NCAM1 has numerous binding partners, few studies have been published about the heterophilic NCAM2 interaction. The extracellular domain of NCAM2 has been shown to bind to Aβ oligomers (42). The FN2 domain of NCAM2 could also bind and activate FGFR1, inducing neurite outgrowth (78). It is therefore possible that the sNCAM2 acts as a dominant negative by binding to membrane-bound NCAM2-FL or other binding partners to NCAM2.

BACE1 expression levels are the highest at the early postnatal stage and then decline from the second postnatal week, reaching relatively low levels in the adult brain (20). NCAM2 expression is detected at E18, reaches maximal levels at P21, and remains at similar levels in adult brain (27). Similarly, NCAM1 protein expression is detected at E13 and observed as a highly polysialylated form in the embryonic and perinatal brains. PSA modification gradually decreases with age, but NCAM1 levels remain stable in the adult brain (27, 60). Thus, we assessed the BACE1-mediated processing of NCAM1 and NCAM2 at three different ages (postnatal day 10, 4- and 12 months) in the OB and the HC. Both NCAM1 and NCAM2 BACE1-derived fragments were detected in the OB of BACE1+/+ at all ages analyzed. In contrast, NCAM2 processing was similar in the HC of BACE1+/+ and BACE1−/− mice, indicating lack of BACE1 cleavage. A significant decrease in sNCAM2β levels and sNCAM1β levels was not associated with a detectable increase in NCAM2-FL and NCAM1-FL levels in OB and HC of BACE1−/− mice. Given that mRNA levels of *Adam10* increase with age (61), unchanged levels of NCAM2-FL and NCAM1-FL in BACE1−/− mice most likely are the results of a compensatory increased processing by ADAM10. However, increased levels of full-length NCAM1 were detected in hippocampal synaptosomes from BACE1−/− mice at 4 months of age, indicating that BACE1 selectively cleaves synaptic NCAM1 in the HC of adult mice. In contrast to NCAM1 and NCAM2, three well-characterized BACE1 substrates (SEZ6, APP, CHL1) were processed by BACE1 in the HC and OB at all ages analyzed, as well as in the hippocampal synaptosomes. Our data suggest that BACE1-mediated processing of NCAMs is spatiotemporally regulated. Further studies will be necessary to determine whether the processing by metalloprotease occurs at times and in regions of the brain different from ones where BACE1 cleaves NCAMs.

Interestingly, we observed increased levels of PSA-NCAM1 in BACE1−/− mouse brain at P10 without a change of total NCAM1-FL levels. Next, we demonstrated that PSA-NCAM1 is cleaved by BACE1 similarly to non-PSA-NCAM1 *in vitro*. The mechanisms leading to increased PSA-NCAM1 in the absence of BACE1 remain to be clarified.

NCAM1 has six glycosylation sites, and two sites in the fifth Ig (Ig5) are polysialylated (Fig. 1*C*)

A single sialic acid molecule is enzymatically added to complex N-glycan in a α-2,3 or α-2,6 linkage by beta-galactoside alpha-2,3-sialyltransferase (ST3Gal) or beta-galactoside alpha-2,6-sialyltransferase (ST6Gal), respectively (28). Then, sialic acid molecules are added to α-2,3 or α-2,6-linked mono-sialylated NCAM1 by ST8SIA2 and ST8SIA4 (29). ST8SIA2 is mainly responsible for PSA modification of NCAM1 in the developing brain, whereas ST8SIA4 is the major polysialyltransferase of the adult brain (30, 31, 32, 33).

Previous proteomic and *in vitro* studies identified beta-galactoside alpha-2,3-sialyltransferase 1 (ST3Gal1) and beta-galactoside alpha-2,6-sialyltransferase 1 (ST6Gal1) as putative BACE1 substrates (14, 79, 80, 81). The coexpression of BACE1 and ST6Gal1 in COS or HEK cells increased the secretion of ST6Gal1 but decreased the levels of ST6Gal1 and 2,6-sialylation in the cells (80). It is likely that BACE1 deletion produces the opposite effect. Therefore, the increased levels of PSA-NCAM1 in BACE1−/− mice at P10 could be the consequence of increased cellular levels of ST3Gal1 and ST6Gal1, resulting in an increased number of α-2,3 or α-2,6-linked mono-sialylated NCAM1 molecules, which in turn can be PSA modified by ST8SIA2 and/or ST8SIA4.

PSA-NCAM1 plays a key role in structural and synaptic plasticity during development and in the adult brain (34, 37, 74). The negatively charged PSA is highly hydrated, thus PSA-NCAM1 has a larger volume than NCAM1 resulting in an increased distance between cells (for review see [82]). Specifically, PSA inhibits NCAM1-mediated homophilic and heterophilic interactions with CAMs, receptors, and ECMs (74, 83). Through this mechanism, PSA-NCAM1 enables the migration of neuronal progenitors and regulates their differentiation during development. Moreover, PSA-NCAM1 is essential for axonal targeting by preventing the formation of aberrant synapses (for review see [82]). Thus, increased postnatal levels of PSA-NCAM1, as observed in BACE1−/− p10 mice, may interfere with axonal targeting.

At the developmental stage, mossy fibers preferentially project to both the stratum lucidum and stratum oriens of the CA3 region of the hippocampus, forming suprapyramidal bundles (SPB) and infrapyramidal bundles (IPB), respectively (84). Previous studies demonstrated that NCAM1 is essential for correct axonal growth and fasciculation of mossy fibers by showing altered distribution of mossy fibers in the hippocampus of NCAM1 knockout mice (57, 58). The abnormal lamination of mossy fibers is also observed in ST8SIA2 knockout mice or in wild-type mice after PSA removal with endo-N treatment, suggesting that PSA modification is critical for the appropriate innervation of mossy fibers (85, 86). Interestingly, NCAM1 knockout and ST8SIA2 knockout mice exhibited extended IPB up to the CA3a subregion (58, 85). This ectopic mossy fiber projection is most likely caused by lack of retraction of misguided axons at the stratum oriens of the CA3. In contrast, a shortened IPB and disorganized mossy fiber bundles were observed in BACE1−/− mice and in CHL1−/− mice (19, 87, 88). Although the mechanism leading to disorganization of the IPB in BACE1−/− mice has not been elucidated, BACE1-mediated processing of CHL1 was hypothesized to be required for proper axon guidance in the dentate gyrus. However, increased PSA-NCAM1 levels in the hippocampus during development could result in enhanced retraction of misguided axons at the stratum oriens and contribute to the defects in mossy fiber targeting observed in BACE1−/− mice. Notably, BACE1 elevation and colocalization with PSA-NCAM are associated with aberrant limbic axonal sprouting in a model of temporal lobe epilepsy (89).

NCAM1-deficient mice showed impaired long-term potentiation (LTP) in CA1 (90, 91) and CA3 (92). However, conditional deletion of NCAM1 in postmitotic glutamatergic forebrain neurons results in impaired LTP in CA1, but not in CA3. These findings suggest that abnormal LTP in CA3 of NCAM1 knockout mice is probably caused by abnormal mossy fiber lamination during brain development (93), which is absent in NCAM1 conditional knockout (cKO) mice. Impaired LTP in the CA1 was also detected in wild-type mice after enzymatic removal of PSA using endo-*N*-acylneuraminidase (Endo-N) treatment and in ST8SIA4 KO mice, indicating that PSA-NCAM1 is required for CA1 LTP (90, 94). Intriguingly, impaired LTP in CA1 of NCAM1 KO mice could be restored by application of PSA only (100 μg/ml) or soluble PSA-NCAM1, but not by high concentration of PSA (300 μg/ml) or non-PSA NCAM1, suggesting that (1) LTP in CA1 is PSA-dependent and that (2) there is optimal PSA concentration for normal LPT in CA1 (95). Interestingly, abnormal LTP in CA1 and CA3 (96, 97) was also found in BACE1−/− mice. Whether an almost complete conditional deletion of BACE1 in adult mice leads to CA1 LTP deficit is controversial. Hu *et al*. (98) reported impairment of LTP in CA1 of conditional BACE1-deficient mice, whereas CA1 LTP was reported to be normal in a different BACE1 cKO mouse model (99). However, we showed that CA1 LTP was impaired in BACE1 cKO mice with a partial reduction of BACE1 (∼50% to ∼70%) (100). Thus, BACE1-dependent imbalance of PSA-NCAM1 could be the underlying mechanism of the abnormal LTP in CA1 in BACE1−/− and BACE1 cKO mice. Other BACE1 substrates may play a role on synaptic plasticity in CA1. However, L1 germline and cKO mice do not show alteration in CA1 LTP. CHL1 germline KO mice show CA1 LTP deficits in juvenile and adult mice but not in young adult mice but data from cKO mice are lacking (for review see [101]). SEZ6 is almost exclusively processed by BACE1 in mice and is required for normal dendritic arborization. SEZ6 null mice display reduced spine density and increased dendritic branching in cortical neurons (102). Germline SEZ6 KO mice show impaired CA1 LTP (103). However, it remains to be established whether conditional deletion of SEZ6 in postmitotic glutamatergic forebrain neurons results in impaired LTP in CA1 as reported for NCAM1.

Taken together, we report here for the first time that NCAM1 and NCAM2 are BACE1 substrates *in vivo*. Our results show that BACE1 differentially processed NCAM1 and NCAM2 depending on the region of the brain and subcellular localization *in vivo*. Whereas NCAM2 does not appear to be cleaved by BACE1 in the HC, NCAM1 is processed in synaptosomes of adult mice. The function of NCAM1 in the brain has been extensively studied. NCAM1 is essential for the plasticity of the developing and adult brain (82). Many of the phenotypes described for BACE1 germline and cKO mice have been also observed in NCAM1 germline and cKO mice. Changes in levels of proteolytic product of NCAM1 and other adhesion molecules have been reported in cerebrospinal fluid and serum from patients with AD and psychiatric disorders (39). Most likely the worsening of cognitive function and other adverse effects including the onset of psychiatric symptoms that caused the interruption of clinical trials with BACE inhibitors (9, 10, 11, 12, 13) are the results of interference with the function of more than one BACE1 substrate. However, given the central role of NCAM1 in brain plasticity, lack of BACE1 cleavage of NCAM1 could represent a major contribution to the mechanism-based side effects of BACE inhibition.

## Experimental procedures

### Expression vectors

The BACE1-GFP expression vector was a gift from Dr Tae Wan Kim (104). pcDNA3.1 vector (Thermo Fisher Scientific) was used as an empty vector. To characterize the BACE1-mediated cleavage of NCAM2 and NCAM1 *in vitro*, expression plasmid containing C-terminally Myc-DDK-tagged mouse transmembrane NCAM2 (MR226626) and NCAM1-140 (MR227176) was purchased from ORIGENE. For polysialic acid modification of NCAM1, expression plasmid containing C-terminally turboGFP tagged mouse ST8SIA2 (MG205823) was purchased from ORIGENE.

### Site-directed mutagenesis of NCAM1 and NCAM2

Mutations on NCAM2 and NCM1 were generated using QuickChange II XL site-directed mutagenesis kit (Agilent) according to the manufacturer's instruction. The mutation was introduced with the following primer (NCAM2-D693A, 5′-CCAAAACCCAACATTATTAAAGCCACACTTTTTAATGGCCTTGGC-3′; NCAM2-D693K, 5′-CATGCCCCCAAAACCCAACATTATTAAAAAGACACTTTTTAATGGCCT-3′; NCAM2-D693H, 5′-CATGCCCCCAAAACCCAACATTATTAAACACACACTTTTTAATG-3′; NCAM2-E663H, 5′-AGTGGACAATGGGCTACCATGTGCAAATCACAGCTGC-3′; NCAM2-E663K, 5′-AGTGGACAATGGGCTACAAAGTGCAAATCACAGCT-3′; NCAM2-YE662/663AH, 5′-CTGCAGTGGACAATGGGCGCCCATGTGCAAATCACAGCTGCC-3′; NCAM2-YE662/663VK, 5′-TCTGCAGTGGACAATGGGCGTCAAAGTGCAAATCACAGCTGC-3′; NCAM1-E671H, 5′-GGACTGGAACGCAGAGTATCATGTCTATGTGGTAGCTGAA-3′; NCAM1-YE670/671AH, 5′-TCCCTGGACTGGAACGCAGAGGCTCATGTCTATGTGGTAGCTGAAAAC-3′; NCAM1-E671K, 5′-GACTGGAACGCAGAGTATAAAGTCTATGTGGTAGCT-3′; NCAM1-YE670/671VK, 5′-CCCTGGACTGGAACGCAGAGGTTAAAGTCTATGTGGTAGCTGAA-3′, along with reverse complement primers). Mutagenesis was confirmed by DNA sequencing.

### Cell culture and transfection

HEK cells (HEK 293T/17; ATCC) used were cultured in Dulbecco's modified Eagles medium (DMEM) supplemented with 10% of fetal bovine serum (FBS) and 1% of penicillin/streptomycin (10,000 U/ml, Thermo Fisher Scientific). HEK cells were seeded at a density of 300,000 per well in six-well plates. When confluency was about 70%, NCAM2-Myc-DDK (0.5 μg) or NCAM1-Myc-DDK (0.5 μg) was cotransfected with BACE1-GFP (0.5 μg) or pcDNA3.1 empty vector using lipofectamine 2000 (Thermo Fisher Scientific) in Opti-MEM Medium (Thermo Fisher Scientific) according to manufacturer's instruction. Mutant NCAM2 (0.5 μg) or NCAM1 (0.5 μg) was used to validate the BACE1 cleavage site of NCAM2 or NCAM1, respectively. TurboGFP-tagged ST8SIA2 (0.5 μg) vector was used for studying BACE1 processing of PSA-NCAM1. Total DNA concentration was kept constant at 1.5 μg with empty vector when necessary. Six hours after transfection, the media were replaced with fresh Opti-MEM Medium containing DMSO (1:1000) or the following inhibitors. All inhibitors were dissolved in DMSO and used at the indicated final concentration for 24 or 48 h: C3, β-secretase inhibitor IV (10 μM, MilliporeSigma); the ADAM10-specific inhibitor GI254023X (5 μM, MilliporeSigma), GM6001, a broad-spectrum inhibitor of matrix metalloproteinases (2.5 μM, MilliporeSigma); DAPT, γ-secretase complex inhibitor (5 μM; STEMCELL Technologies). Conditioned media were collected, supplemented with 1X protease and phosphatase inhibitor cocktail (Thermo Fisher Scientific), and cleared by centrifugation at 14,000*g* for 30 min. Cells were washed with cold DPBS buffer, then 200 μl of RIPA (radioimmunoprecipitation assay) buffer (150 mM NaCl, 1% NP-40, 0.5% sodium deoxycholate, 0.1% SDS, 1 mM EDTA, and 50 mM Tris HCl, pH 7.4) containing 1X protease and phosphatase inhibitor cocktail (Thermo Fisher Scientific) was added. Collected cell lysates were homogenized by hand homogenizer and sonicated briefly. After incubation in 4 °C for 30 min, cell lysates were harvested after centrifugation at 14,000*g* for 20 min. Protein concentrations were determined using BCA assay (Thermo Fisher Scientific). Adjusted volume of conditioned media for equal amount of total protein of cell lysates was used to analyze the secreted protein.

### Mice handling

Wild-type mice (C57BL/6J; Stock No: 000664) and BACE1−/− mice (BACE1KO; Stock No: 004714) on a C57BL/6J background were purchased from the Jackson Laboratories. All mice were maintained on a 12-h light–dark cycle, and food and water were available ad libitum. All animal experiments were performed with the approval of Tufts University Institutional Animal Care and Use Committees.

### Serial fractionation of mouse brain samples

Male and female mice were anesthetized with isoflurane, and the hippocampus and olfactory bulbs were rapidly dissected from the mouse brain and snap-frozen in liquid nitrogen. After weighing the snap-frozen mouse hippocampi or olfactory bulb, samples were homogenized in ten volumes of PBS buffer containing 1X protease inhibitor and 1X phosphatase inhibitor cocktail with 30 strokes on a Dounce homogenizer. PBS homogenates were ultracentrifuged at 100,000*g* for 1 h to obtain PBS soluble fraction (soluble). The remaining pellet was washed with PBS buffer and spun at 100,000*g* for 5 min. After removing the supernatant, remaining pellet was lysed in ten volumes of RIPA buffer, sonicated, rotated for 30 min at 4 °C, and then ultracentrifuged at 100,000*g* for 1 h. The supernatant was collected and then stored in −80 °C as membrane fraction (membrane). Protein concentrations were determined using BCA assay (Thermo Fisher Scientific).

### Immunoprecipitation

To identify BACE1 cleavage site of NCAM1 and NCAM2, HEK cells were transfected with NCAM2 (MR226626) or NCAM1-140 (MR227176) expression plasmid with BACE1-GFP plasmid using lipofectamine 2000 as explained above. After 24–48 h posttransfection, cells were washed with cold DPBS buffer and lysed with RIPA buffer containing 1X protease and phosphatase inhibitor cocktail. After preclearing with 50 μl of protein A/G PLUS-agarose beads (sc-2003; Santa Cruz Biotechnology) by incubation for 1 h at 4 °C, NCAM1 (NCAM1-FL and NCAM1- βCTFs) or NCAM2 (NCAM1-FL/NCAM2-βCTFs) proteins were immunoprecipitated from the cell lysates using 8 μl of anti-Myc antibody (9B11; Cell Signaling) and 80 μl of protein A/G PLUS-agarose beads in 500 μl of lysis buffer overnight at 4 °C. After washing the beads with PBS, proteins were diluted with sample buffer and boiled at 95 °C for 5 min. All the samples were used for mass spectrometry analysis.

### Mass spectrometry analysis

After electrophoresis of immunoprecipitated samples, SDS-polyacrylamide gel electrophoresis gels were stained with QC Colloidal Coomassie Strain (1610803, Bio-Rad). Stained individual bands (NCAM2-βCTF, NCAM2-FL, NCAM1-βCTF, and NCAM1-FL) were excised from the gel and submitted for microcapillary tandem liquid chromatography tandem mass spectrometry (LC/MS/MS) to the Taplin Mass Spectrometry Facility, Harvard Medical School, Boston, MA, as previously described (105). Excised gel bands were cut into approximately 1 mm^3^ pieces. Gel pieces were then subjected to a modified in-gel trypsin digestion procedure (106). Gel pieces were washed and dehydrated with acetonitrile for 10 min followed by removal of acetonitrile. Pieces were then completely dried in a speed-vac. Rehydration of the gel pieces was with 50 mM ammonium bicarbonate solution containing 12.5 ng/μl modified sequencing-grade trypsin (Promega, Madison, WI) at 4 °C. After 45 min, the excess trypsin solution was removed and replaced with 50 mM ammonium bicarbonate solution to just cover the gel pieces. Samples were then placed in a 37 °C room overnight. Peptides were later extracted by removing the ammonium bicarbonate solution, followed by one wash with a solution containing 50% acetonitrile and 1% formic acid. The extracts were then dried in a speed-vac (∼1 h). The samples were then stored at 4 °C until analysis. Samples were reconstituted in 5–10 μl of HPLC solvent A (2.5% acetonitrile, 0.1% formic acid). A nanoscale reverse-phase HPLC capillary column was created by packing 2.6 μm C18 spherical silica beads into a fused silica capillary (100 μm inner diameter × ∼30 cm length) with a flame-drawn tip (107). After equilibrating the column each sample was loaded *via* a Famos auto sampler (LC Packings) onto the column. A gradient was formed, and peptides were eluted with increasing concentrations of solvent B (97.5% acetonitrile, 0.1% formic acid). As peptides eluted, they were subjected to electrospray ionization and then entered into an LTQ Orbitrap Velos Pro ion-trap mass spectrometer (Thermo Fisher Scientific). Peptides were detected, isolated, and fragmented to produce a tandem mass spectrum of specific fragment ions for each peptide. Peptide sequences (and hence protein identity) were determined by matching protein databases with the acquired fragmentation pattern by the software program, Sequest (version 28, rev. 13, Thermo Fisher Scientific) (108). Fixed modification of 57.0214 Da on cysteine (iodoacetamide) and a variable modification of 15.9949 Da on methionine (oxidation) were considered. Searches were performed using a 50 ppm precursor ion tolerance. Fragment ion tolerance was set to 1 Da. All databases include a reversed version of all the sequences and the data was filtered to between a 1% and 2% peptide false discovery rate.

### Isolation of synaptosome

Protocol for synaptosome isolation (109, 110) was modified as follows. After weighing the snap-frozen mouse, the hippocampus was homogenized in ten volumes of homogenate buffer (HB; 0.32 M Sucrose, 20 mM HEPES, 1 mM EDTA, pH 7.4). The homogenates were centrifuged at 1000*g* for 10 min to remove nuclear and cell debris. Supernatant (S1) was saved, and the pellet was resuspended in six volumes of HB and spun again at 1000*g* for 10 min. After spinning, the supernatant (S2) was combined with previous supernatants (S1) and then centrifuged at 13,000*g* for 20 min. The pellet was washed with HB and spun again at 13,000*g* for 20 min. The resulting crude synaptosomal pellet was resuspended in ten volumes of HB and subsequently loaded on the top of sucrose step gradient (1.2 M sucrose on the lower, 0.8 M sucrose on the middle, and crude synaptosome on the top). Sucrose was prepared in 20 mM HEPES, 1 mM EDTA, and pH 7.4. After ultracentrifugation at 100,000*g* for 2 h, the synaptosomal fraction was collected at the 0.8/1.2 M interface, diluted with HB, and pulled down by ultracentrifugation at 100,000*g* for 1 h. Resulting purified synaptosomal pellet was lysed in six volumes of 2% Triton-RIPA buffer, sonicated, and rotated for 30 min at 4 °C. After centrifugation at 14,000*g* for 20 min at 4 °C, the supernatant was collected for immunoblot analysis. Protein concentrations were determined using BCA assay (Thermo Fisher Scientific). All the steps were performed at 4 °C, and all the buffers were supplemented with complete protease and phosphatase inhibitor cocktail (Thermo Fisher Scientific).

### Removal of sialic acid modification on NCAM1

Soluble or membrane fraction of HC and OB from BACE1+/+ and BACE1−/− mice was incubated with or without the α2-3,6,8,9 Neuraminidase A (Sialidase; P0722; New England Biolabs) in a reaction solution, which is provided by the manufacturer, to remove the sialic acid from NCAM1 proteins at 37 °C overnight.

### BACE1 enzymatic activity on PSA-NCAM1 or non-PSA-NCAM1

Hemibrain at P10 was homogenized in ten volumes of RIPA buffer containing 1X protease inhibitor and 1X phosphatase inhibitor cocktail. NCAM1. After preclearing with 50 μl of protein A/G PLUS-agarose beads (sc-2003; Santa Cruz Biotechnology) by incubation for 1 h at 4 °C, total NCAM1 was immunoprecipitated using 50 μl of anti-C-terminal NCAM1 antibody (5B8-s; DSHB) and 80 μl of protein A/G-agarose beads in 300 μl of P10 lysates overnight at 4 °C. After washing the beads with PBS, the beads were incubated with or without the α2-3,6,8,9 Neuraminidase A (Sialidase; P0722; New England Biolabs) in a reaction solution, which is provided by the manufacturer, to remove sialic acid from purified NCAM1 proteins at 37 °C overnight. After washing the beads with PBS, PSA-NCAM1 or non-PSA-NCAM1 proteins were sequentially incubated in a reaction buffer (100 mM sodium acetate, pH 4.5) including recombinant human BACE1 (931-AS-050; R&D systems) at 37 °C overnight. For positive control of BACE1 processing, immunopurified endogenous APP proteins with anti-APP antibody (C1/6.1; BioLegend) were incubated with recombinant human BACE1 in a reaction buffer at 37 °C overnight.

### Western blot analysis

Prepared samples (5∼30 μg) with sample buffer were generally boiled at 70 °C for 10 min and separated on 4–12% Bis-Tris gels (Bio-Rad) in XT MES or XT MOPS running buffers (Bio-Rad). While 10% Bis-Tris gels (Bio-Rad) with XT MES running buffer were used to separate NCAM1-CTFs or NCAM2-CTFs, 16.5% Tris-Tricine gels (Bio-Rad) with Tris/Tricine/SDS running buffer (Bio-Rad) were used to separate the APP-CTFs. SeeBlue Plus2 Pre-stained Protein Standard (Thermo Fisher Scientific) was used for Figures 2 and 3 and Figure S2. Precision Plus Protein Dual Color Standards (Bio-Rad) was used for all other figures. Proteins were transferred onto polyvinylidene difluoride (PVDF) membrane and blocked for 1 h in 5% nonfat dried milk in TBST. The membrane was then incubated overnight at 4 °C with the following primary antibodies. anti-NCAM2 (mouse mAb, 1:1000; sc-136328; Santa Cruz), anti-NCAM2 (goat pAb, 1:6000; GTX89311; GeneTex), anti-NCAM1 (goat pAb, 1:2000; AF2408; R&D systems), anti-NCAM1 (mouse mAb, 1:400; clone 5B8; DSHB), anti-NCAM1 (mouse mAb, 1:4000; clone 0B11; MilliporeSigma), anti-NCAM1 (rabbit pAb, 1:8000; AB5032; MilliporeSigma), anti-PSA-NCAM1 (mouse mAb; 1:2000;clone 2-2B; MilliporeSigma), anti-Myc (rabbit pAb, 1:1000; 2272; Cell Signaling), anti-turboGFP (mouse mAb, 1:2000; clone OTI2H8; ORIGENE), anti-SEZ6 (rat mAb, 1:250; clone 14E5; kindly provided by Dr S. Lichtenthaler) (24) and anti-sAPPβ (mouse mAb, 1:40; clone BAWT kindly provided by Dr S. Lichtenthaler) (111), anti-β-tubulin (rabbit pAb, 1:1000; 2146; Cell Signaling), anti-BACE1 (rabbit mAb, 1:1500; D10E5; Cell signaling); anti-CHL1 (goat pAb, 1:2000; AF2147; R&D systems); anti-calnexin (mouse mAb, 1:1000; 610523; BD biosciences); anti-GAPDH (mouse mAb, 1:10,000; MAP374; Millipore), anti-PSD95 (rabbit pAb, 1:1500, ab18258, Abcam), and anti-synaptophysin 1 (mouse mAb, 1:1000, 101011; Synaptic System). Primary antibodies were detected with the species specific horseradish peroxidase (HRP)-conjugated secondary antibody and then visualized by ECL. Chemiluminescent signal was captured on an LAS4000 Fuji Imager. Densitometry analysis was performed using Quantity One (Bio-Rad) or Multi Gauge (GE) software.

### Immunohistochemistry

After perfusion with PBS, collected hemibrain for histological analysis was post fixed in 4% paraformaldehyde in PBS for 24 h and cryoprotected in 30% sucrose in PBS containing 0.02% sodium azide. Sagittal sections (40 μm) were cut on a sliding microtome and collected in cryoprotectant (30% sucrose, 1% Polyvinyl-pyrrolidone (PVP-40), 0.05 M phosphate buffer, 30% ethylene glycol), and then processed for histological analysis. Sections were washed and treated with 0.1 M sodium citrate pH 9 for 30 min for antigen retrieval. Sections were then blocked in 5% BSA in 0.25% triton-X 100 (TBS-T) for 2 h. After washing in 1% BSA in TBS-T, sections were incubated with primary antibodies (mouse anti-BACE1; 1:250; clone 3D5 kindly provided by Dr R. Vassar, rabbit anti-NCAM1; 1:200; AB5032, goat anti-NCAM2; 1:200; AF778; R&D systems) in 1% BSA in TBS-T overnight at 4 °C, followed by secondary antibodies (anti-mouse Alexa 647, anti-rabbit Alexa 568, and anti-goat Alexa 488) in 1% BSA in TBS-T for 2 h at room temperature. Sections were mounted on Superfrost slides (Thermo Fisher Scientific) using Fluoromount-G1 mounting media (Southern Biotech), and Z-stack fluorescence images were obtained using confocal microscopes (SP8, SPE TCS Leica) fitted with an HC PL APO CS2 20x oil lenses.

### Statistical analysis

Data are expressed as mean ± standard deviation, represented as error bars. One-way analysis of variance (ANOVA) with Tukey's multiple comparison test was performed to evaluate statistical difference among the groups. Unpaired two-tailed *t*-test with Welch's correction was used for statistical comparison between two groups. *p* < 0.05 was considered statistically significant for all the experiments. Statistical analysis was performed using the GraphPad Prism Software.

## Data availability

The mass spectrometry proteomics data have been deposited to the ProteomeXchange Consortium *via* the PRIDE (112) partner repository with the data set identifier PXD021330.

## Supporting information

This article contains supporting information.

## Conflict of interest

The authors declare that they have no conflicts of interest with the contents of this article.
