# Supplementary material for: Spatiotemporal processing of neural cell adhesion molecules 1 and 2 by BACE1 in vivo
Source: J Biol Chem. 2021 Feb 3;296:100372. doi: 10.1016/j.jbc.2021.100372 (PMC7949136; doi:10.1016/j.jbc.2021.100372)
Supplement: Figures S1 to S9 and Table S1 [file mmc1.pdf]

Supporting Information

Spatiotemporal processing of neural cell adhesion molecules 1 and 2 by BACE1 *in vivo*

WonHee Kim, Hiroto Watanabe, Selene Lomoio, Giuseppina Tesco

Alzheimer's Disease Research Laboratory, Department of Neuroscience, Tufts University School of Medicine, 136 Harrison Avenue, Boston, MA 02111, USA

*Corresponding author: Giuseppina Tesco

Email: Giuseppina.Tesco@Tufts.edu

Running Title: BACE1 differentially processes NCAM1 and NCAM2 *in vivo*

List:

Figure S1. Identification of BACE1 specific N-terminal fragment of NCAM2 and NCAM1 in olfactory bulb.

Figure S2. Identification of BACE1 specific C-terminal fragment of NCAM1 in olfactory bulb.

Figure S3. NCAM1 and NCAM2 are co-localized with BACE1 in the olfactory bulb.

Figure S4. Full length version of the western blots shown in Figure 2C (A) and Figure 3C (B).

Figure S5. Identification of multiple C-terminal fragments of NCAM1 in HEK cells.

Figure S6. Peptides of full-length NCAM2-TM (A) and 32-kDa NCAM2-βCTF (B) identified from mass spectrometry analysis were mapped to the protein sequences.

Figure S7. Peptides of full-length NCAM1-140 (C) and 38-kDa NCAM1-βCTF (D) identified from mass spectrometry analysis were mapped to the protein sequences.

Figure S8. BACE1 does not cleave NCAM2 at D693.

Figure S9. Removal of polysialic acid from NCAM1.

Table S1. List of the NCAM1 and NCAM 2 antibodies used in this study.

Table S2. List of all peptides identified from mass spectrometry analysis (excel file).

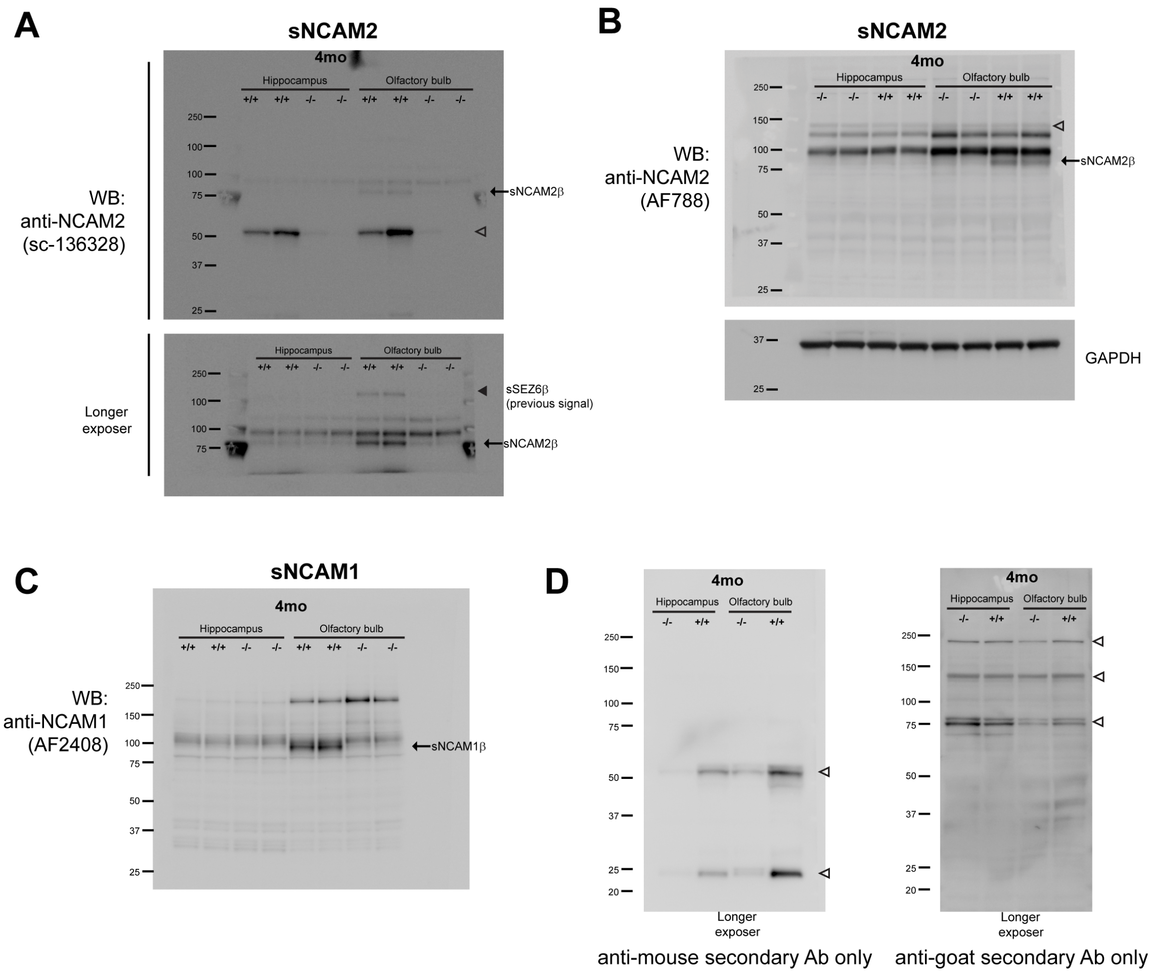

Figure S1. Identification of BACE1 specific N-terminal fragment of NCAM2 and NCAM1 in olfactory bulb. A-C, PBS soluble fractions of the hippocampus and olfactory bulb samples from 4-months-old (4mo) BACE1+/+ and BACE1-/- mice were immunoblotted with anti-NCAM2 (sc-136328) (A), anti-NCAM2 (AF778) (B), anti-GAPDH (MAB374) (B), and anti-NCAM1 (AF-2408) (C) antibodies. BACE1-specific soluble NCAM2 (sNCAM2β) and NCAM1 (sNCAM1β) were observed in the olfactory bulb of BACE1+/+ mice, but not in BACE1-/- mice. A and C, full-length versions of the western blots (sNCAM2β and sNCAM1β) of 4-months-old mice shown in Figure 5A. After longer exposer, weak bands (arrowhead in A) are observed, which correspond to previously immunoblotted sSEZ6β. D, western blot with HRP-conjugated secondary antibody (anti-mouse and anti-goat) without primary antibody incubation produced non-specific bands (open arrowhead). However, these non-specific bands (open arrowhead in A and B) do not correspond to sNCAM2β or sNCAM1β.

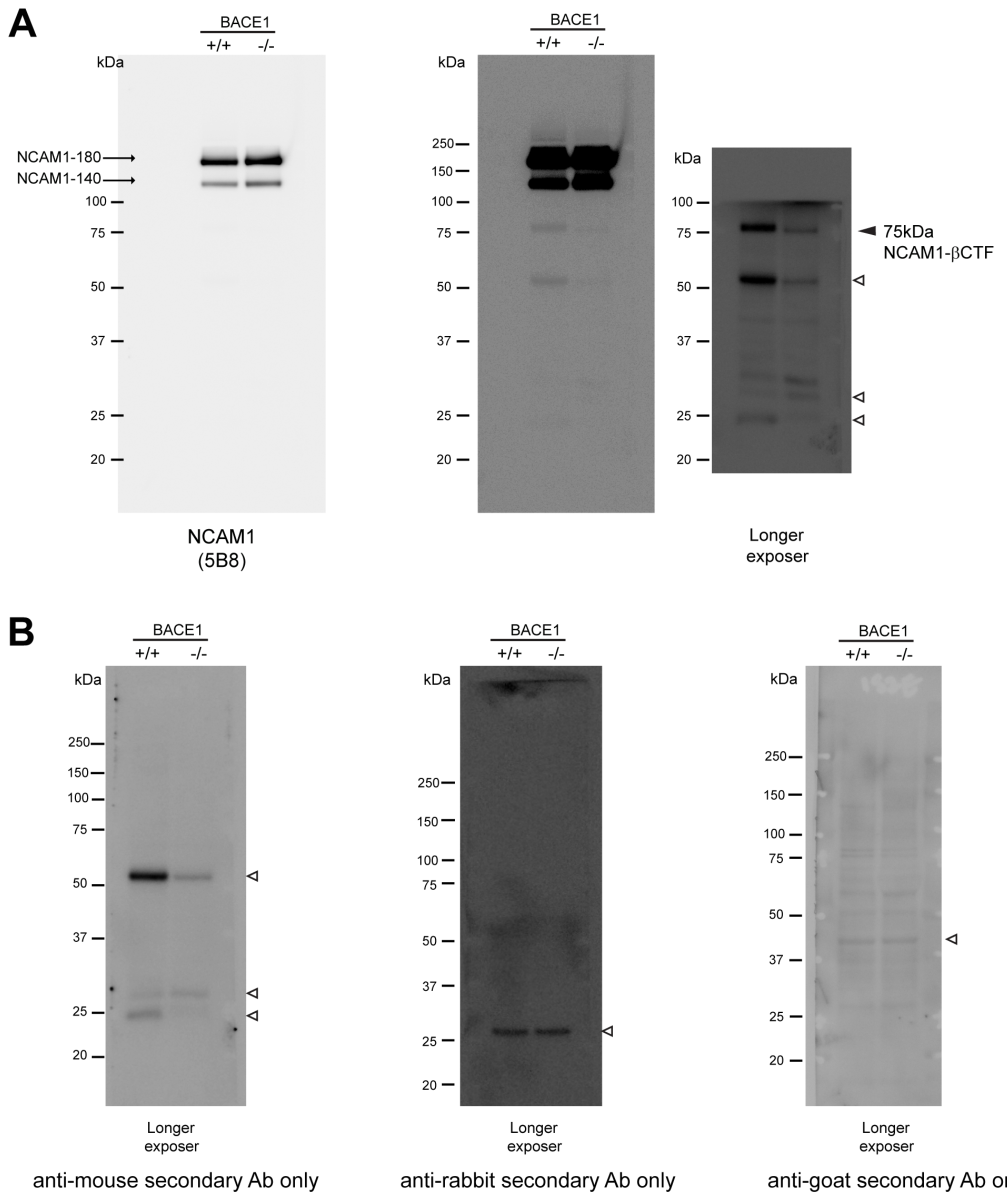

Figure S2. Identification of BACE1 specific C-terminal fragment of NCAM1 in olfactory bulb. A, representative immunoblot of membrane fractions of olfactory bulb samples from 4-month-old BACE1+/+ and BACE1-/- mice using anti-C-terminal NCAM1 (5B8) antibody. After longer exposure with the upper part of the blot covered to block the strong signal from NCAM1-140 and NCAM1-180 full-length, a ~75 kDa NCAM1-CTF was detected in BACE1+/+ mice, but it was greatly decreased in BACE1-/- mice, and thus termed NCAM1-βCTF (arrowhead). Other bands at ~65kDa and ~25kDa (open arrowhead) are non-specific bands. BACE1+/+; n=3, BACE1-/-; n=3. B, HRP-conjugated secondary antibody (anti-mouse, anti-rabbit, and anti-goat) without primary antibody incubation produced non-specific bands (open arrowhead). However, these non-specific bands (open arrowhead in A) are not the same band of NCAM1-βCTF at 75kDa.

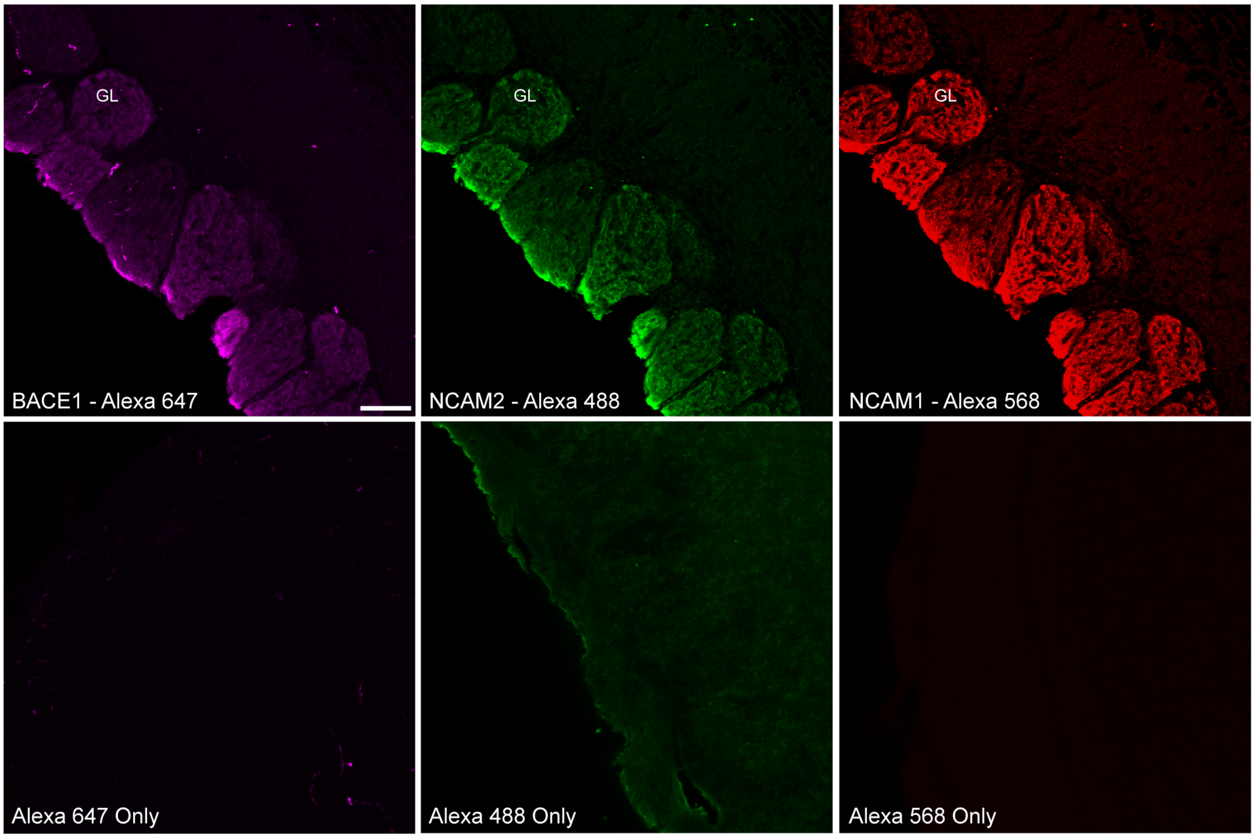

Figure S3. NCAM1 and NCAM2 are co-localized with BACE1 in glomeruli (GL) of the olfactory bulb. Top panel: coronal section from 12-month-old BACE1+/+ olfactory bulb was stained with anti-BACE1 (3D5 with Alexa 647, magenta), anti-NCAM2 (AF778 with Alexa 488, green), and anti-NCAM1 (AB5032 with Alexa 568, red) antibodies. Bottom panel: secondary antibodies were only applied on the adjacent OB coronal sections in order to verify the staining specificity of each antibody. No secondary antibody background was detectable for the Alexa 568 and 647 antibodies and only some diffuse and nonspecific signal was present after incubation with Alexa 488. The secondary antibody only images have been acquired by increasing the laser gain by approximately 10% in order to collect any eventual residual signal. Z-stack confocal images. Magnification 20x. Digital Zoom 1.15. Scale bar represents 100 um, n=2.

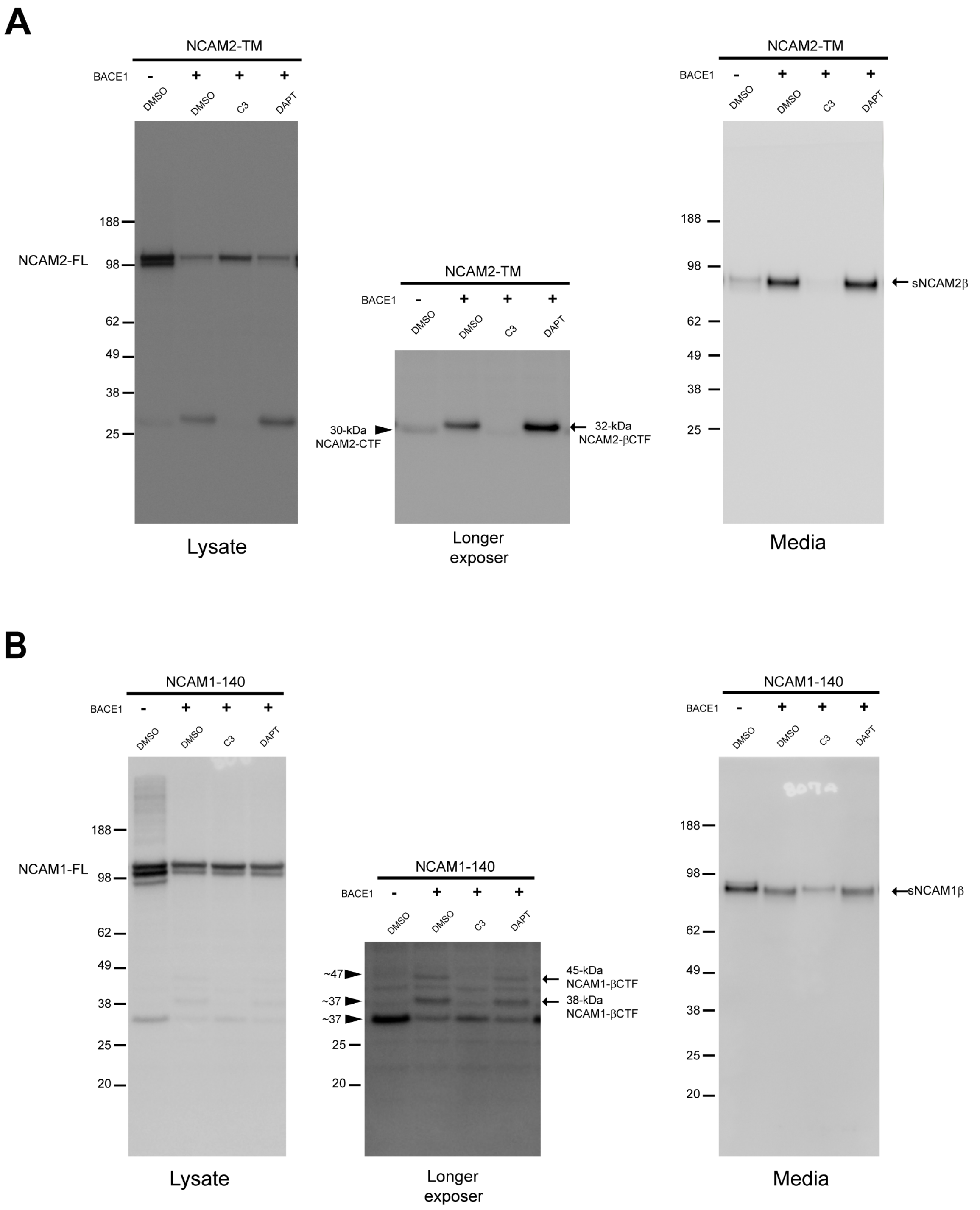

Figure S4. Full-length versions **of western blots shown in Figure 2C (A) and Figure 3C (B).** During a longer exposure to better detect the CTFs, the upper part of the blot was covered to block the strong signal from NCAM1 and NCAM 2 full-length.

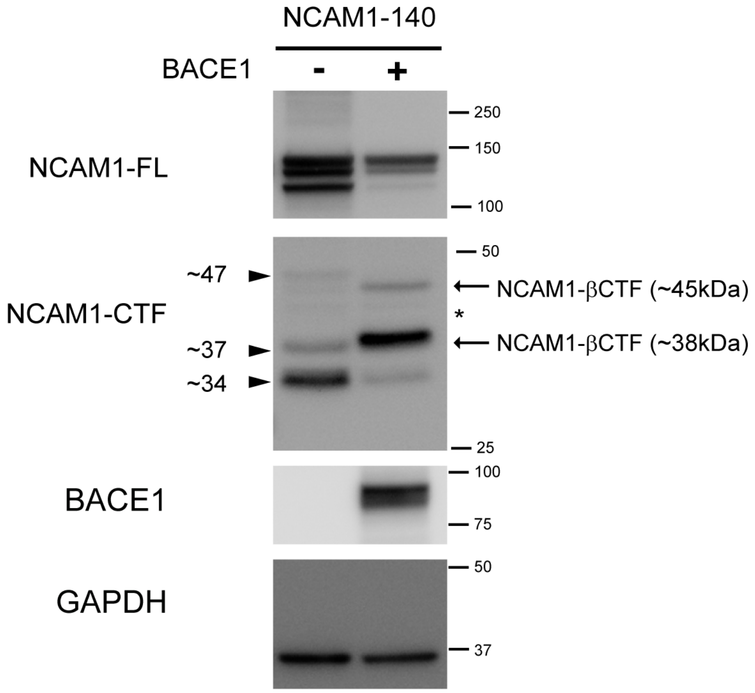

Figure S5. Identification of multiple C-terminal fragments of NCAM1 in HEK cells. In order to clearly separate and identify NCAM1-CTFs, we compared 10% Bis-Tris gel (3450112; Bio-Rad) (Fig. S1) and 4~12% Bis-Tris gel (3450124; Bio-Rad) (used for Fig. 2 and Fig. 3). Representative immunoblot of cell lysates using anti-Myc (2272), anti-BACE1 (D10E5) and anti-GAPDH (MAB374) antibodies. 10% Bis-Tris gel shows the three bands of NCAM1-CTFs (~34kDa, ~37kDa, and ~47kDa) and two bands of NCAM1-βCTFs (~38kDa and ~45kDa) more clearly than 4~12% Bis-Tris gel. Also, a non-specific band (asterisk) is not well detected by the 10% Bis-Tris gel compared to 4~12% Bis-Tris gel (see Fig. 3A and 3C)

A **NCAM2-FL**

```
  1 MSLLLSFYLL GLLVRSGQAL LQVTISLSKV ELSVGESKFF TCTAIGEPES
 51 IDWYNPQGEK IISTQRVMLQ KEGVRSRLTI YNANIEDAGI YRCQATDAKG
101 QTQEATVVLE IYQKLTFREV VSPQEFKQGE DAEVVCRVSS SPAPAVSWLY
151 HNEEVTTIPD NRFAVLANNN LQILNINKSD EGIYRCEGRV EARGEIDFRD
201 IIVIVNVPPA IMMPQKSFNA TAERGEEMTL TCKASGSPDP TISWFRNGKL
251 IEENEKYILK GSNTELTVRN IINKDGGSYV CKATNKAGED QKQAFLQVFV
301 QPHILQLKNE TTSENGHVTL VCEAEGEPVP EITWKRAIDG VMFSEGDKSP
351 DGRIEVKGQH GRSSLHIRDV KLSDSGRYDC EAASRIGGHQ RSMHLDIEYA
401 PKFVSNQTMY YSWEGNPINI SCDVTANPPA SIHWRREKLL LPAKNTTHLK
451 THSVGRKMIL EIAPTSDNDF GRYNCTATNR IGTRFQEYIL ELADVPSSPH
501 GVKIIELSQT TAKISFNKPE SHGGVPIHHY QVDVKEVASE TWKIVRSHGV
551 QTMVVLSSLE PNTTYEIRVA AVNGKGQGDY SKIEIFQTLP VREPSPPSIH
601 GQPSSGKSFK ISITKQDDGG APILEYIVKY RSKDKEDQWL EKKVQGNKDH
651 IILEHLQWTM GYEVQITAAN RLGYSEPTVY EFSMPPKPNI IKDTLFNGLG
701 LGAIIGLGVA ALLLILVVTD VSCFFIRQCG LLMCITRRMC GKKSGSSGKS
751 KELEEGKAAY LKDGSKEPIV EMRTEDERIT NHEDGSPVNE PNETTPLTEP
801 EKLPLKEENG KEVLNAETIE IKVSNDIIQS KEDDIKA
```

B **32-kDa NCAM2-βCTF**

```
  1 MSLLLSFYLL GLLVRSGQAL LQVTISLSKV ELSVGESKFF TCTAIGEPES
 51 IDWYNPQGEK IISTQRVMLQ KEGVRSRLTI YNANIEDAGI YRCQATDAKG
101 QTQEATVVLE IYQKLTFREV VSPQEFKQGE DAEVVCRVSS SPAPAVSWLY
151 HNEEVTTIPD NRFAVLANNN LQILNINKSD EGIYRCEGRV EARGEIDFRD
201 IIVIVNVPPA IMMPQKSFNA TAERGEEMTL TCKASGSPDP TISWFRNGKL
251 IEENEKYILK GSNTELTVRN IINKDGGSYV CKATNKAGED QKQAFLQVFV
301 QPHILQLKNE TTSENGHVTL VCEAEGEPVP EITWKRAIDG VMFSEGDKSP
351 DGRIEVKGQH GRSSLHIRDV KLSDSGRYDC EAASRIGGHQ RSMHLDIEYA
401 PKFVSNQTMY YSWEGNPINI SCDVTANPPA SIHWRREKLL LPAKNTTHLK
451 THSVGRKMIL EIAPTSDNDF GRYNCTATNR IGTRFQEYIL ELADVPSSPH
501 GVKIIELSQT TAKISFNKPE SHGGVPIHHY QVDVKEVASE TWKIVRSHGV
551 QTMVVLSSLE PNTTYEIRVA AVNGKGQGDY SKIEIFQTLP VREPSPPSIH
601 GQPSSGKSFK ISITKQDDGG APILEYIVKY RSKDKEDQWL EKKVQGNKDH
651 IILEHLQWTM GYEVQITAAN RLGYSEPTVY EFSMPPKPNI IKDTLFNGLG
701 LGAIIGLGVA ALLLILVVTD VSCFFIRQCG LLMCITRRMC GKKSGSSGKS
751 KELEEGKAAY LKDGSKEPIV EMRTEDERIT NHEDGSPVNE PNETTPLTEP
801 EKLPLKEENG KEVLNAETIE IKVSNDIIQS KEDDIKA
```

Figure S6. Peptides of full-length NCAM2-TM (A) and 32-kDa NCAM2-βCTF (B) identified (blue) from mass spectrometry analysis were mapped to the protein sequences.

A **NCAM1-FL**

```
  1 MLRTKDLIWT LFFLGTAVSL QVDIVPSQGE ISVGESKFFL CQVAGDAKDK
 51 DISWFSPNGE KLSPNQQRIS VVWNDDDSST LTIYNANIDD AGIYKCVVTA
101 EDGTQSEATV NVKIFQKLMF KNAPTPQEFK EGEDAVIVCD VVSSLPPTII
151 WKHKGRDVIL KKDVRFIVLS NNYLQIRGIK KTDEGTYRCE GRILARGEIN
201 FKDIQVIVNV PPTVQARQSI VNATANLGQS VTLVCDADGF PEPTMSWTKD
251 GEPIENEEED DEKHIFSDDS SELTIRNVDK NDEAEYVCIA ENKAGEQDAS
301 IHLKVFAKPK ITYVENQTAM ELEEQVTLTC EASGDPIPSI TWRTSTRNIS
351 SEEKTLDGHM VVRSHARVSS LTLKSIQYTD AGEYICTASN TIGQDSQSMY
401 LEFQYAPKLQ GPVAVYTWEG NQVNITCEVF AYPSATISWF RDGQLLPSSN
451 YSNIKIYNTP SASYLEVTPD SENDFGNYNC TAVNRIGQES LEFILVQADT
501 PSSPSIDRVE PYSSTAQVQF DEPEATGGVP ILKYKAEWKS LGEESWHFKW
551 YDAKEANMEG IVTIMGLKPE TRYSVRLAAL NGKGLGEISA ATEFKTQPVR
601 EPSAPKLEGQ MGEDGNSIKV NLIKQDDGGS PIRHYLVKYR ALASEWKPEI
651 RLPSGSDHVM LKSLDWNAEY EVYVVAENQQ GKSKAAHFVF RTSAQPTAIP
701 ANGSPTAGLS TGAIVGILIV IFVLLLVVMD ITCYFLNKCG LLMCIAVNLC
751 GKAGPGAKGK DMEEGKAAFS KDESKEPIVE VRTEEERTPN HDGGKHTEPN
801 ETTPLTEPEK GPVETKSEPP ESEAKPAPTE VKTVPNDATQ TKENESKA
```

B **38-kDa NCAM1-βCTF**

```
  1 MLRTKDLIWT LFFLGTAVSL QVDIVPSQGE ISVGESKFFL CQVAGDAKDK
 51 DISWFSPNGE KLSPNQQRIS VVWNDDDSST LTIYNANIDD AGIYKCVVTA
101 EDGTQSEATV NVKIFQKLMF KNAPTPQEFK EGEDAVIVCD VVSSLPPTII
151 WKHKGRDVIL KKDVRFIVLS NNYLQIRGIK KTDEGTYRCE GRILARGEIN
201 FKDIQVIVNV PPTVQARQSI VNATANLGQS VTLVCDADGF PEPTMSWTKD
251 GEPIENEEED DEKHIFSDDS SELTIRNVDK NDEAEYVCIA ENKAGEQDAS
301 IHLKVFAKPK ITYVENQTAM ELEEQVTLTC EASGDPIPSI TWRTSTRNIS
351 SEEKTLDGHM VVRSHARVSS LTLKSIQYTD AGEYICTASN TIGQDSQSMY
401 LEFQYAPKLQ GPVAVYTWEG NQVNITCEVF AYPSATISWF RDGQLLPSSN
451 YSNIKIYNTP SASYLEVTPD SENDFGNYNC TAVNRIGQES LEFILVQADT
501 PSSPSIDRVE PYSSTAQVQF DEPEATGGVP ILKYKAEWKS LGEESWHFKW
551 YDAKEANMEG IVTIMGLKPE TRYSVRLAAL NGKGLGEISA ATEFKTQPVR
601 EPSAPKLEGQ MGEDGNSIKV NLIKQDDGGS PIRHYLVKYR ALASEWKPEI
651 RLPSGSDHVM LKSLDWNAEY EVYVVAENQQ GKSKAAHFVF RTSAQPTAIP
701 ANGSPTAGLS TGAIVGILIV IFVLLLVVMD ITCYFLNKCG LLMCIAVNLC
751 GKAGPGAKGK DMEEGKAAFS KDESKEPIVE VRTEEERTPN HDGGKHTEPN
801 ETTPLTEPEK GPVETKSEPP ESEAKPAPTE VKTVPNDATQ TKENESKA
```

Figure S7. Peptides of full-length NCAM1-140 (C) and 38-kDa NCAM1-βCTF (D) identified (blue) from mass spectrometry analysis were mapped to the protein sequences.

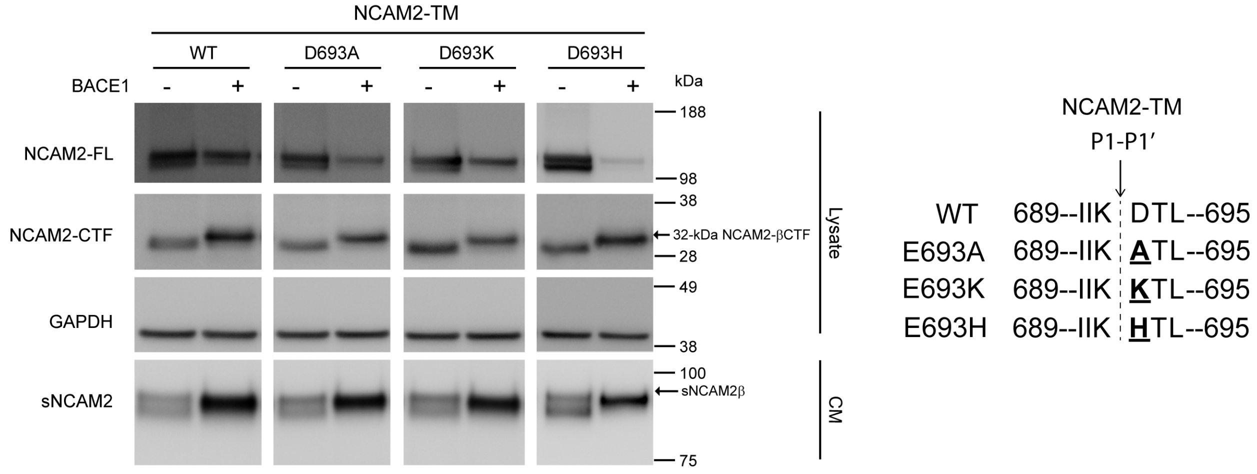

Figure S8. BACE1 does not cleave NCAM2 at D693. Wild type (WT) or mutant NCAM2 (E693A, D693K, D693H) were co-transfected with an empty vector or BACE1 into the HEK cells and cell lysates were analyzed by immunoblot to assess the BACE1 processing of NCAM2 at Asp 693 ($I^{691}K^{692} \downarrow D^{693}T^{694}$; The ↓ symbol denotes the scissile bond). Representative immunoblot of cell lysates (Lysate) and conditioned media (CM) using anti-Myc (2272), anti-NCAM2 (sc-136328), anti-BACE1 (D10E5) and anti-GAPDH (MAB374) antibodies. None of these NCAM2 mutations at D693 did not prevent the production of BACE1-specific 32kDa NCAM2-βCTF in the cell lysates and sNCAM2β in the CM. Note that a different protein ladder was used in this figure compared to Fig. 1. n=3.

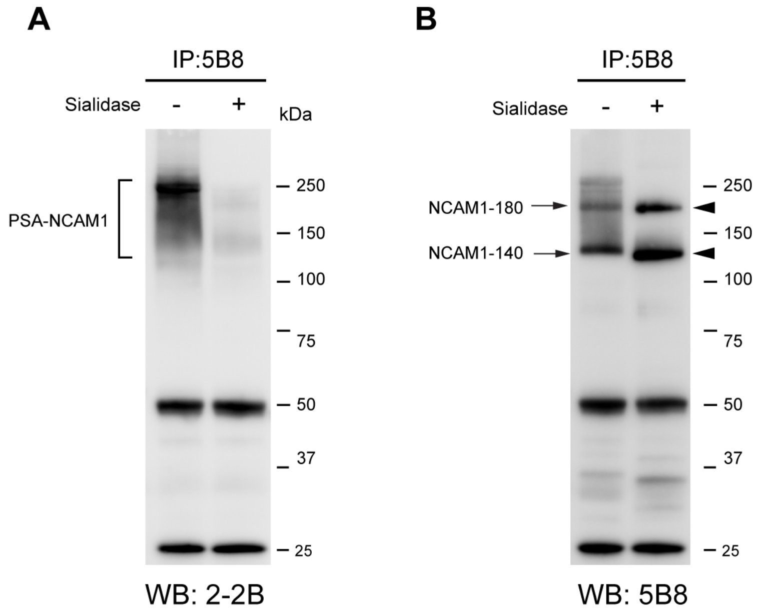

Figure S9. Removal of polysialic acid from NCAM1. NCAM1 proteins from P10 hemibrain lysates were immunoprecipitated using anti-C-terminal NCAM1 antibody (5B8). Given that PSA modification on NCAM1 is enriched at P10, NCAM1/5B8-bead complex was incubated in a reaction buffer with or without sialidase (P0722) to remove sialic acid modification from NCAM1. After washing the beads with PBS, immunoprecipitated NCAM1 proteins with or without sialidase treatment were analyzed by western blot. A, immunoblot analysis with anti-PSA-NCAM1 (2-2B) antibody confirmed the removal of polysialic acid on NCAM1 by sialidase enzyme. B, after removal of PSA on NCAM1, the intensity of two bands, representing non-PSA-NCAM1 (NCAM1-180 and NCAM1-140), were stronger than the two bands detected from immunoprecipitated NCAM1 proteins without sialidase, owing to the increased non-PSA-NCAM1 by sialidase treatment. Note that the molecular weight of the two bands (arrowheads), representing non-PSA-NCAM1 (NCAM1-180 and NCAM1-140), was slightly lower than the corresponding PSA-NCAM1-180 and -140 due to the removal of sialic acid by sialidase.

Table S1. List of the NCAM1 and NCAM 2 antibodies used in this study.

Antibody	Host species	Epitope	Application in reference	Validated in KO tissue (Yes / No)	Reference
NCAM2 (AF778)	Goat	aa 20-700	WB, IHC	Yes	(1)
NCAM2 (sc-136328)	Mouse	aa 478-677	WB, IHC, IP, IC	No	(11,12)
NCAM2 (GTX89311)	Goat	C-terminal (aa 822-836)	PL, IC	No	(13)
NCAM1 (AF2408)	Goat	aa 20-603	WB, IHC	No	(14,15)
NCAM1 (5B8)	Mouse	C-terminal	WB	Yes	(2)
NCAM1 (AB5032)	Rabbit	C-terminal	WB, IHC	Yes and conditional KO mice	(3,4)
NCAM1 (0B11)	Mouse	C-terminal	WB, IHC	No	(16,17)
PSA-NCAM1 (2-2B)	Mouse	PSA	WB, IHC	Yes and endo-N treated wild type mice	(4)

IHC; Immunohistochemistry, WB; Western blot, IC; Immunocytochemistry, PL; proximity ligation analysis

References

1. Deleyrolle, L., Sabourin, J. C., Rothhut, B., Fujita, H., Guichet, P. O., Teigell, M., Ripoll, C., Chauvet, N., Perrin, F., Mamaeva, D., Noda, T., Mori, K., Yoshihara, Y., and Hugnot, J. P. (2015) OCAM regulates embryonic spinal cord stem cell proliferation by modulating ErbB2 receptor. *PLoS One* **10**, e0122337
2. Leshchyns'ka, I., Sytnyk, V., Morrow, J. S., and Schachner, M. (2003) Neural cell adhesion molecule (NCAM) association with PKCbeta2 via betaI spectrin is implicated in NCAM-mediated neurite outgrowth. *J Cell Biol* **161**, 625-639
3. Bukalo, O., Fentrop, N., Lee, A. Y., Salmen, B., Law, J. W., Wotjak, C. T., Schweizer, M., Dityatev, A., and Schachner, M. (2004) Conditional ablation of the neural cell adhesion molecule reduces precision of spatial learning, long-term potentiation, and depression in the CA1 subfield of mouse hippocampus. *J Neurosci* **24**, 1565-1577
4. Watzlawik, J. O., Kahoud, R. J., Ng, S., Painter, M. M., Papke, L. M., Zoecklein, L., Wootla, B., Warrington, A. E., Carey, W. A., and Rodriguez, M. (2015) Polysialic acid as an antigen for monoclonal antibody HIgM12 to treat multiple sclerosis and other neurodegenerative disorders. *J Neurochem* **134**, 865-878
5. Zhao, J., Fu, Y., Yasvoina, M., Shao, P., Hitt, B., O'Connor, T., Logan, S., Maus, E., Citron, M., Berry, R., Binder, L., and Vassar, R. (2007) Beta-site amyloid precursor protein cleaving enzyme 1 levels become elevated in neurons around amyloid plaques: implications for Alzheimer's disease pathogenesis. *J Neurosci* **27**, 3639-3649
6. Kim, W., Ma, L., Lomoio, S., Willen, R., Lombardo, S., Dong, J., Haydon, P. G., and Tesco, G. (2018) BACE1 elevation engendered by GGA3 deletion increases beta-amyloid pathology in association with APP elevation and decreased CHL1 processing in 5XFAD mice. *Mol Neurodegener* **13**, 6
7. Kuhn, P. H., Koroniak, K., Hogl, S., Colombo, A., Zeitschel, U., Willem, M., Volbracht, C., Schepers, U., Imhof, A., Hoffmeister, A., Haass, C., Rossner, S., Brase, S., and Lichtenthaler, S. F. (2012) Secretome protein enrichment identifies physiological BACE1 protease substrates in neurons. *EMBO J* **31**, 3157-3168
8. Pigoni, M., Hsia, H. E., Hartmann, J., Rudan Njavro, J., Shmueli, M. D., Muller, S. A., Guner, G., Tushaus, J., Kuhn, P. H., Kumar, R., Gao, P., Tran, M. L., Ramazanov, B., Blank, B., Hipgrave Ederveen, A. L., Von Blume, J., Mulle, C., Gunnersen, J. M., Wuhrer, M., Rammes, G., Busche, M. A., Koeglsperger, T., and Lichtenthaler, S. F. (2020) Seizure protein 6 controls glycosylation and trafficking of kainate receptor subunits GluK2 and GluK3. *EMBO J*, e103457
9. Kotarska, A., Fernandes, L., Kleene, R., and Schachner, M. (2020) Cell adhesion molecule close homolog of L1 binds to the dopamine receptor D2 and inhibits the internalization of its short isoform. *FASEB J* **34**, 4832-4851
10. Barão, S., Gärtner, A., Leyva-Díaz, E., Demyanenko, G., Munck, S., Vanhoutvin, T., Zhou, L., Schachner, M., López-Bendito, G., Maness, P. F., and De Strooper, B. (2015) Antagonistic Effects of BACE1 and APH1B-γ-Secretase Control Axonal Guidance by Regulating Growth Cone Collapse. *Cell reports* **12**, 1367-1376
11. Sheng, L., Leshchyns'ka, I., and Sytnyk, V. (2015) Neural cell adhesion molecule 2 promotes the formation of filopodia and neurite branching by inducing submembrane increases in Ca2+ levels. *J Neurosci* **35**, 1739-1752
12. Leshchyns'ka, I., Liew, H. T., Shepherd, C., Halliday, G. M., Stevens, C. H., Ke, Y. D., Ittner, L. M., and Sytnyk, V. (2015) Abeta-dependent reduction of NCAM2-mediated synaptic adhesion contributes to synapse loss in Alzheimer's disease. *Nat Commun* **6**, 8836
13. Sheng, L., Leshchyns'ka, I., and Sytnyk, V. (2018) Neural Cell Adhesion Molecule 2 (NCAM2)-Induced c-Src-Dependent Propagation of Submembrane Ca2+ Spikes Along Dendrites Inhibits Synapse Maturation. *Cerebral cortex (New York, N.Y. : 1991)*
14. Pistritto, G., Papaleo, V., Sanchez, P., Ceci, C., and Barbaccia, M. L. (2012) Divergent modulation of neuronal differentiation by caspase-2 and -9. *PLoS One* **7**, e36002

15. Birkhoff, J. C., Brouwer, R. W. W., Kolovos, P., Korporaal, A. L., Bermejo-Santos, A., Boltsis, I., Nowosad, K., van den Hout, M., Grosveld, F. G., van, I. W. F. J., Huylebroeck, D., and Conidi, A. (2020) Targeted chromatin conformation analysis identifies novel distal neural enhancers of ZEB2 in pluripotent stem cell differentiation. *Hum Mol Genet* **29**, 2535-2550
16. Yoshihara, Y., Kawasaki, M., Tamada, A., Fujita, H., Hayashi, H., Kagamiyama, H., and Mori, K. (1997) OCAM: A new member of the neural cell adhesion molecule family related to zone-to-zone projection of olfactory and vomeronasal axons. *J Neurosci* **17**, 5830-5842
17. Abe, C., Yi, Y., Hane, M., Kitajima, K., and Sato, C. (2019) Acute stress-induced change in polysialic acid levels mediated by sialidase in mouse brain. *Scientific reports* **9**, 9950
